# An integrative taxonomic approach reveals unexplored diversity in Croatian planarians

**DOI:** 10.1186/s12983-026-00603-8

**Published:** 2026-03-16

**Authors:** Miquel Vila-Farré, Jeremias N. Brand, Tobias Boothe, Maren Brockmeyer, Fruzsina Ficze-Schmidt, Markus A. Grohme, Uri Weill, Kasper H. Kluiver, Ludwik Gąsiorowski, Lucija Kauf, Yuliia Kanana, Helena Bilandžija, Marta Riutort, Jochen C. Rink

**Affiliations:** 1 Department of Tissue Dynamics and Regeneration, Max Planck Institute for Multidisciplinary Sciences, Göttingen, Germany; 2 Max Planck Institute of Molecular Cell Biology and Genetics, Dresden, Germany; 3 Computing, Mathematics, Engineering and Natural Sciences Faculty, Northeastern University London, London, UK; 4 Institute of Evolutionary Biology, Faculty of Biology, University of Warsaw, Warsaw, Poland; 5 Ruđer Bošković Insititute, Bijenička Cesta 54, Zagreb, Croatia; 6 Institute of Neurogenetics, University of Lübeck, Lübeck, Germany; 7 I.I. Schmalhausen Institute of Zoology, National Academy of Sciences of Ukraine, Kiev, Ukraine; 8 Departament de Genètica, Microbiologia i Estadística, Universitat de Barcelona, Barcelona, Spain; 9 Institut de Recerca de la Biodiversitat (IRBio), Universitat de Barcelona, Barcelona, Spain; 10 Faculty of Biology and Psychology, Georg-August-University Göttingen, Göttingen, Germany

**Keywords:** Diversity, Planarians, Integrative taxonomy, Croatia, Barcoding, *Cytochrome c oxidase subunit I*, *Dendrocoelum pigmentatum*

## Abstract

**Background:**

Freshwater ecosystems are among the most endangered habitats on Earth, with approximately one-fourth of aquatic species at risk of extinction. Effective conservation efforts require comprehensive monitoring and accurate species identification, including often overlooked groups. Planarian flatworms are one such group that, although commonly present in freshwater ecosystems worldwide, remains understudied even in species-rich areas, e.g. Croatia. As a result, the true extent of planarian diversity often remains underappreciated.

**Results:**

With the goal of characterising the Croatian planarian diversity, we used an integrative approach combining barcoding and classical taxonomy methods. Motivated by the highly skewed representation of planarian diversity in current GenBank records of the barcoding gene *cytochrome c oxidase subunit I* (COI), we first optimised primer design and amplification protocols. Applying these approaches to field-collected material from Croatia, we substantially expanded the number of taxonomically curated COI barcode sequences for European dugesiids, dendrocoelids and planariids. In addition, our efforts resulted in the description of a new pigmented *Dendrocoelum* species, *Dendrocoelum pigmentatum* Vila-Farré, sp. nov.*,* the discovery of two highly differentiated haplotypic clades in *Schmidtea lugubris*, and the rediscovery of *Polycladodes alba* in Croatia after a century.

**Conclusions:**

Overall, our effort integrates Croatia as an underexplored but planaria species-rich region into the endeavour to systematically describe the planarian fauna of Europe. The expansion of a known number of Croatian planarian species from eight to sixteen and the discovery of a new, large planarian species in continental Europe, *Dendrocoelum pigmentatum*, demonstrate the effectiveness of our integrative approach. Overall, our work highlights the underappreciated diversity of planarians, even in continental Europe and supports practical conservation efforts to preserve aquatic biodiversity.

**Supplementary Information:**

The online version contains supplementary material available at 10.1186/s12983-026-00603-8.

## Introduction

Assessing organismal diversity is critical amidst the ongoing biodiversity crisis that threatens global ecosystems [1–3]. The decline in vertebrate biodiversity, such as amphibians, is well-documented [4, 5]. In contrast, invertebrates, which contain 99% of animal diversity [6], remain, with few exceptions [7], understudied. As a result, only a small proportion of invertebrates are considered for conservation actions [8]. This knowledge gap is particularly concerning for freshwater ecosystems, which are among Earth's most endangered habitats [9]. Freshwater ecosystems account for the highest proportion of species extinctions and harbour many threatened species, with approximately one-fourth currently at risk of extinction [10]. This figure likely underestimates the crisis's true extent, as many data-deficient groups remain poorly studied [11, 12], underscoring the need for increased research and monitoring [13].

Freshwater planarians (Platyhelminthes, Tricladida) are renowned for their remarkable regenerative capabilities, making species like *Schmidtea mediterranea* and *Dugesia japonica* models in stem cell and regeneration research [14–16]. Recent studies have emphasised the need to incorporate additional planarian species to better understand the evolutionary processes underlying regeneration [17–20]. However, the actual diversity of planarians is poorly understood. This is more striking given their worldwide distribution, abundance, and potential for studying a wide range of biological questions [16, 21–25]. Consequently, the conservation status of many planarians is unknown. The decline of the model species *S. mediterranea* in Spain highlights the need for efforts in planarian conservation [26]. Despite this, the IUCN Red List, a recognised international reference inventory of biological species’ global conservation status and extinction risk [27], lists only two planarian species as assessed in 2024 [12]. One of the reasons is that traditional taxonomy in planarians is carried out via the reconstruction of copulatory apparatus morphology from histological sections, which is time-consuming and requires specialist training and extensive experience [28]. DNA barcoding has emerged as a powerful tool that promises rapid and accessible species identification [29]. The term refers to the sequencing of DNA regions that evolve so rapidly that they tend to accumulate species- or even population-specific single-nucleotide polymorphisms (SNPs). Commonly used barcoding loci include the nuclear ribosomal rRNA genes (e.g. 18S) for genus or species-level identification and the mitochondrial *cytochrome c oxidase subunit I* (COI) locus for species or population-level analyses [30, 31]. In planarians, barcoding species delimitation has been primarily used in dugesiids of the Mediterranean area and land planarians, but rarely so far in other planarian groups [32–36]. Nevertheless, the integration of traditional taxonomy and barcoding methods has substantially improved our knowledge of planarians in the area, especially for dugesiids [37–39], highlighting the potential of this combined approach.

Croatia lies within the Mediterranean biodiversity hotspot [40] and at the intersection of several biogeographic regions [41], with a dynamic geological history contributing to its rich biodiversity. Our review of taxonomically curated records identified at least eight freshwater planarian species in the country, primarily based on studies from the twentieth century [42–50]: *Polycelis felina* (Dalyell, 1814), *Phagocata dalmatica* (Stanković & Komárek, 1927), *Crenobia alpina* (Dana, 1766), *Crenobia anophtalma* Mrázek, 1907, *Dendrocoelum romanodanubiale* (Codreanu, 1949), *Dendrocoelum subterraneum* Komárek, 1919, *Polycladodes alba* Steinmann, 1910, and *Dugesia absoloni* (Komárek, 1919). Records of planarian species that are not taxonomically documented are not included in this list (e.g. [51, 52]. This relatively low species count suggests that Croatia's freshwater planarian diversity is likely underestimated, particularly when considered in conjunction with the higher species richness of other Mediterranean regions, including the Iberian Peninsula, Sardinia and the Aegean islands, which have been investigated in recent years using a combination of molecular and classical methods [37, 39, 53, 54].

To address this potential gap, we surveyed freshwater planarians in Croatia’s karstic region. Our study integrates DNA barcoding, RNA sequencing, and traditional taxonomic methods, including histological analyses and metaphase chromosome preparations. Our findings expand the number of known Croatian planarian species from eight to sixteen, including the discovery and description of a new, large and pigmented dendrocoelid species. In addition, we advance the utility of barcoding for planarian taxonomy by providing a robust protocol and new barcode sequences for previously unstudied planarian clades, thus generally facilitating species identification and monitoring in this understudied group.

## Methods

### Field collections

The specimens analysed in this study were collected via several dedicated field expeditions to Croatia and other parts of Europe between 2013 and 2023. Planarians were sampled from the underside of stones, aquatic plants or other submerged objects with the help of a soft brush to minimise damage to the animals [20]. After collection, specimens were transferred into 50 ml conical bottom tubes, with care taken to maintain low animal densities to prevent worm lysis. The tubes were kept cool, and water from the collection site was used for daily water changes during the field campaign to maintain health and viability. Each collection site’s GPS coordinates and detailed habitat characteristics were recorded in a dedicated database for future reference.

### Live imaging

Flatworms were imaged either in the field or under laboratory conditions. Field imaging was conducted using a Canon EOS 6D Mark II camera equipped with a Canon EF 100 mm f/2.8L Macro IS USM lens and a Canon Extension Tube EF25 II to allow for a shorter focal distance. Two Walimex Pro LED Strip Light Slim 300 Daylights were used for illumination. Laboratory imaging was performed using a ZEISS Stereo Microscope Stemi 508 paired with a ZEISS Axiocam 208 colour digital camera for detailed morphological documentation.

### Sample preparations, imaging and analysis of histological sections

Specimens for morphological studies were euthanised under laboratory conditions using nitric acid solution (4.8 M HNO_3_, 300 mM NaCl in Milli-Q water) followed by fixation in 10% formalin solution (4% methanol stabilised-formaldehyde, 44.5 mM Na_2_HPO_4_ in Milli-Q water) for 2 days at room temperature (RT). The nitric acid solution formulation is the same as for the planarian fixative Steinmann's fluid [55], except that it excludes mercuric chloride (HgCl₂). To prepare specimens, living individuals were placed in a 10 cm Petri dish containing a small volume of planarian water (PW) [56] or water from their habitat and allowed to stretch out. Once the specimens were fully stretched, nitric acid solution was poured over them and left for 30 s to 1 min. After removing the liquid, the specimens were rinsed with 10% formalin solution and transferred to 10% formalin solution for two days at RT. Finally, they were transferred to 70% ethanol in Milli-Q water using a soft brush or plastic pipette. The ethanol was replaced 2–3 times to ensure proper preservation. Samples were stored at RT. Toward the end of the project, the formalin step was eliminated from the protocol and samples euthanised with nitric acid solution were rinsed once in 1 × phosphate-buffered saline (PBS) and directly transferred to 70% ethanol in Milli-Q water.

For histological sections, fixed specimens were dehydrated in a graded ethanol series, cleared in clove oil to facilitate the screening for the presence or absence of a copulatory apparatus, and embedded in Paraplast Plus in a Leica TP1020 automatic tissue processor. Serial sections at intervals of 5 µm were made and stained with Mallory-Cason/Heidenhain stain [55]. The stained sections were imaged with an Olympus VS200 widefield slide scanner with an Olympus UPLXAPO20X objective. Reconstructions of the copulatory apparatus were drawn manually on the basis of representative images using the Affinity Designer software and an XP-PEN Artist 15.6 Pro tablet. All processed slides will be deposited in the collections of the newly forming Biodiversitätsmuseum of Göttingen, Göttingen, Germany (ZMUG code) and the Croatian Natural History Museum, Zagreb, Croatia.

### COI NCBI search

All flatworm COI sequences present in the NCBI nucleotide archive on 2024-04-11 were retrieved using the following search term: “(((Platyhelminthes[Organism]) AND (CO1[Gene Name] OR COX1[Gene Name] OR COXI[Gene Name] OR COI[Gene Name] OR cytochrome oxidase subunit I[Title] NOT environmental[Title])))”, including their taxonomic ID. The list of taxonomic IDs was further processed using TaxonKit (v0.18.0) [57] and plotted in R [58].

### COI degenerate primer design

For designing primers for planarian COI, PlanMine transcriptomes [59, 60] and publicly available COI planarian sequences were aligned using Geneious [61]. The species used for primer design included the families Dugesiidae (*Schmidtea mediterranea*, *Schmidtea polychroa*), Planariidae (*Planaria torva*, *Polycelis tenuis*, *Polycelis nigra*) and Dendrocoelidae (*Dendrocoelum lacteum).* The alignments were manually inspected for highly conserved regions suitable for primer design. Potential primer sequences were analysed using PerlPrimer [62] for potential hairpin formation and tuned in length for PCR annealing temperature optimisation. Primer positions with a degeneracy of three or fewer were included as equimolar wobble bases. To reduce the complexity of the primer mixture, higher degeneracy positions were replaced with the universal base inosine, which allows pairing with any base [63]. Therefore, *Taq* polymerase is required for PCR amplification with the MVCOI900 primer pair, as proofreading polymerases, such as *Pfu,* cannot utilise inosine-containing primers [64]. The MVCOI900 primer pair yields an ~ 880 bp amplicon that can be directly sequenced via Sanger sequencing (no need for cloning; see Additional file 1).

Genomic DNA as template for the COI barcoding PCR reactions was extracted with a modification of the previously described protocol [65] (see “Rapid DNA isolation protocol”). The protocol combines a guanidinium thiocyanate-based lysis buffer (GTC buffer) with a phenol–chloroform extraction. Either freshly cut pieces of live planarians or individuals fixed in 100% ethanol were used as starting material. The demuconization step in the original protocol was omitted. The lysis buffer was supplemented with 10% v/v 1 M DTT right before use instead of 7% v/v β-mercaptoethanol. Phase-maker gel tubes were replaced by 2 ml microcentrifuge tubes. Lysis and precipitation were done at RT. The pellet was washed twice with 1 ml 70% ethanol to remove salts.

Individual COI gene sequences were obtained by PCR amplification. The final volume of each PCR reaction was 50 µl, including 2 µl of genomic DNA (1–20 ng), 5 µl of *Taq* Standard buffer, 2 µl of MgCl_2_ (25 mM), 1 µl of dNTPs (10 mM), 1.5 µl of each primer (10 µM), 0.25 µl of *Taq* polymerase (NEB) (Cat. No M0273S), and 36.75 µl of nuclease-free water.

Amplification conditions were as follows: (1) 5 min at 95 °C, (2) 30 s at 95 °C, (3) 30 s at 50 °C, (4) 1 min at 68 °C, and (5) 3 min at 68 °C. Steps 2, 3, and 4 were repeated for 30 cycles. Amplification products were analysed by gel electrophoresis (1% agarose gel) and purified using the QIAquick PCR Purification Kit (Cat. No 28104). The purified amplification products were sequenced in both directions using the amplification primers. Complementary sequencing reads were assembled using Geneious [61] and translated into amino acids to ensure reading frame integrity.

For comparing the amplification efficiency of the MVCOI900 primer pair with published primers, the widely used BarT/COIR primer pair [66] was selected due to its similar amplicon size (Additional file 2). The PCR amplification conditions described above for the MVCOI900 were applied for the first comparison (Additional file 3A). For the BarT/COIR, the conditions were extracted from the bibliography [66]: (1) 2 min at 95 °C, (2) 50 s at 94 °C, (3) 45 s at 43 °C, (4) 50 s at 68 °C, and (5) 4 min at 68 °C. Steps 2, 3, and 4 were repeated for 30 cycles.

Because both primer pairs exhibited comparable amplification in this first test, samples that failed or amplified weakly were identified (Additional file 3B). These challenging samples were then used in a second comparison (Additional file 3C), in which modified PCR conditions were evaluated (reduced annealing temperature for MVCOI900; addition of BSA and/or DMSO). The experimental conditions for all comparisons are described in Additional file 3.

### COI barcoding

A dataset with 136 COI barcodes (Dataset Barcoding) was compiled for the barcoding analysis. This dataset is composed of sequences generated with the MVCOI900 primers for Croatian samples and for others collected during European lab expeditions (78 sequences), and GenBank COI sequences of European freshwater planarians (58 sequences) (Additional file 4). First, an initial alignment was generated using the software MAFFT (v7.490) [67] (Algorithm G-INS-I; Scoring matrix 200PAM/k = 2; Gap open penalty = 1.53; Offset values = 0.123) implemented in Geneious [61], which resulted in a 1,780 bp alignment with 59% missing data. This is referred to as “*full”* alignment in the text (Additional file 2B). The high proportion of missing data was due to the inclusion of sequences amplified with primers targeting shorter COI fragments, as well as complete COI sequences obtained from mitochondrial genomes, obtained from GenBank (Additional file 2B). To assess the robustness of our inference, we also generated two trimmed alignments with less missing data. The first was created using SeqKit (v0.15.0) [68], retaining only columns with at least 50% occupancy (-gap 50). The second was obtained by manually trimming the alignment ends to include only the region represented by the short barcoding fragment. We refer to these as the “*gap50”* and “*small”*, respectively. The *gap50* alignment was 837 bp long with 17% missing data, while the *small* alignment was 432 bp long with 9% missing data (Additional file 2B). Maximum likelihood (ML) phylogenies were inferred for all three alignments using IQ-TREE [69]), first determining the best substitution model using ModelFinder (‘-m TEST’) and calculating 1000 ultra-fast bootstraps and SH-like approximate likelihood ratio test with 1000 replicates to assess branch support (-bnni -bb 1000 -alrt 1000). Branches with ultra-fast bootstrap values lower than 95 were collapsed using the function “Delete branches” implemented in iTOL [70].

Additionally, Bayesian phylogenies were inferred for the *full* and *gap50* alignments; the *small* alignment was excluded from Bayesian analysis because the ML results indicated it lacked sufficient resolution. For both the *full* and *gap50* alignments, the best-fitting substitution model according to the Bayesian Information Criterion (BIC) was K3Pu + F + I + G4. This model, a variant of the Kimura three-parameter model [71], accounts for unequal transition rates, unequal base frequencies, a proportion of invariable sites (+ I), and rate heterogeneity among sites modelled with a gamma distribution with four categories (+ G4). As this model is not implemented in many Bayesian inference programs, we used RevBayes (v1.3.1) [72], which allows flexible specification of substitution models. For both alignments, we ran four independent MCMC chains with a burn-in of 100,000 generations, sampling every 100th generation. The total chain lengths were 6,713,000 and 8,308,000 generations for the *full* and *gap50* alignments, respectively. Convergence among independent chains was assessed by inspecting trace plots in Tracer (v.1.7.2) [73] and confirming that all model parameters reached an effective sample size (ESS) of at least 625 using the convenience R package (v1.0.0) [74]. Posterior tree distributions from independent runs were concatenated, and maximum clade credibility (MCC) trees were computed using the phangorn R package (v2.8.13) [75]. Cophylogenetic plots were generated using the phytools R package (v1.0–1) [76]. All analyses were performed in R (v4.1.2).

### Haplotype networks

Two sequence datasets were prepared to build haplotype networks and to study the relationships among the different haplotypes of *Schmidtea lugubris* detected in the barcoding analysis. Dataset 1 was composed of 16 COI sequences extracted from GenBank or generated for this study as described above. The resulting alignment contained overlapping sequences with a final length of 308 bp. Dataset 2 was composed of 12 COI sequences exclusively generated in this study. The resulting alignment contained overlapping sequences with a final length of 702 bp. In both cases, ambiguous positions (three in total) were removed from the original alignments before analysis. The aligned sequences were imported into DnaSP6 v.6 [77] to generate Roehl Data files. Roehl Data files were imported into Network v10.2 to calculate haplotype networks using the Median-joining network algorithm [78].

### Metaphasic plate production and analysis

Individuals corresponding to distinct *S. lugubris* haplotypes identified through barcoding were selected for karyotypic comparison. The individuals were cut twice between the root of the pharynx and the eyes. The middle piece was preserved in 100% ethanol for barcoding analysis (see “Barcoding”). The posterior piece was fixed in nitric acid solution for histological studies (see “Sample preparation, imaging, and analysis of histological sections”). The anterior piece was used for karyological analysis. Anterior pieces were placed in 6-well plates containing PW [56] and incubated at 20 °C. PW was replaced twice on the first day. After two days of regeneration at 20 °C, samples were incubated for 6 h in a solution of 400 ng/ml Nocodazole (Sigma, M1404) and 1% DMSO in PW to elicit the metaphase-arrest of dividing cells. Specimens were then fixed in 3:1 methanol:glacial acetic acid and stored at −20 °C for a minimum of 30 min, with some samples stored for several days before further processing.

Upon resuming the protocol, samples were brought to RT. Tissue containing the blastema and post-blastema regions was excised with a surgical razor blade, macerated in 45% glacial acetic acid (diluted in Milli-Q water) for 30 min at RT. The macerated tissue was minced on a glass slide, transferred to a 1.5 ml microcentrifuge tube, and pipetted up and down 10 times to create a cell suspension. A drop of this suspension was placed at the centre of a preheated (65 °C on a hot plate) 15 mm Ø coverslip. After approximately 5 min of drying, dried coverslips were transferred to 12-well plates with the tissue side facing up and stored at 4 °C.

To stain metaphase plates, coverslips were washed with 1 × PBS for 5 min with gentle agitation, incubated for 30 min in a solution of 1.7 µg/ml of 4′,6-diamidino-2-phenylindole (DAPI) in 1 × PBS (protected from light), and washed twice with 1 × PBS and twice with Milli-Q water for 5 min each. Coverslips were gently dried with paper and mounted on glass slides using 10 µl of ProLong™ Glass Antifade Mountant (Thermo, Invitrogen, P36982). Mounted slides were cured for 12 h at RT and stored at 4 °C.

Metaphase plate images were captured using an Olympus VS200 widefield slide scanner equipped with X-Cite XYIS XT720L LED illumination. DAPI was excited using a 352–404 nm bandpass filter, and the fluorescence signal was collected with a 416–452 nm bandpass emission filter. Imaging involved three successive steps. A UPLFLN4X 4 × air objective (NA = 0.13, WD = 17 mm) was used to locate the tissue area. Selected regions were scanned with a UPLXAPO20X 20 × air objective (NA = 0.8, WD = 0.6 mm) to identify chromosome groups visually. High-resolution imaging of individual metaphase plates was performed with a UPLAPO100XOHR 100 × oil objective (NA = 1.5, WD = 0.12), producing image stacks for each plate. Image stacks were processed in FIJI [79] and deconvolved using a theoretical point spread function (Gibson & Lanni 3D Optical Model) [80] for 200 iterations. A deconvolved substack of 1–3 planes was finally maximum projected to generate final metaphase plate images.

Chromosome counts were performed in FIJI. For Nottingham and Black Drim, laboratory populations were used, derived from wild specimens maintained in our planarian collections [20]. Four individuals and 4–7 metaphase plates per individual were analysed. For the Croatian population of *S. lugubris*, five metaphase plates from a single individual were analysed. In total, 54 metaphase plates from 9 individuals were examined. Chromosomes were arranged by relative length (%), calculated as (chromosome length*100)/total length of haploid complement.

### RNA extraction, sequencing, and transcriptome assembly

RNA was extracted from planarian samples using a combination of TRIzol-based homogenisation, chloroform extraction, and commercial column purification. Briefly, for most samples, TRIzol reagent was added to the sample, which was then snap-frozen in liquid nitrogen and thawed on ice. The sample was homogenised using metal beads in the TissueLyser II, and chloroform was added to recover the upper phase. Isopropanol and high-salt-precipitation solution (0.8 M sodium citrate and 1.2 M NaCl in Milli-Q water) were added to the aqueous phase, and the mixture was purified by the Direct-zol RNA MiniPrep (R2072) Kit.

A DNA-RNA combined extraction method was used for two samples (GOE00548 and GOE00576). Samples were homogenised in GTC buffer using the TissueLyser II, and the homogenate was separated into two phases using a phenol–chloroform method. The upper phase was transferred to two new tubes, with 40% of the volume used for DNA extraction and 60% for RNA extraction. DNA samples were treated with *RNase A* and purified using a standard DNA extraction protocol (see above). RNA samples were purified as described above.

RNA quality was assessed using a Bioanalyzer RNA 6000 Nano kit (5067–1511). Double-indexing was used to minimise cross-contamination of transcriptomes. RNA was then processed for 150-base-pair paired-end Illumina sequencing, performed by the Dresden Concept Genome Center and Azenta Life Sciences.

Adapters, low complexity reads, and low-quality bases were removed from the short reads using fastp (v = 0.23.4, ‘-l -c −5 5 -M 30 -r 5 -l 35 –detect_adapter_for_pe’) [81]. Then, Kraken 2 (v = 2.1.3) [82] was used with the standard database and default parameters in combination with KrakenTools [83] (‘extract_kraken_reads.py –exclude –taxid 2 2157 10,239 –include-children’) to filter out potential contamination from viruses, bacteria, and archaea. De novo transcriptome assemblies were generated from the filtered reads using Trinity (v = 2.15.2, ‘–SS_lib_type RF ‘) [84].

Potentially contaminating contigs originating from the bovine liver food or the experimenters themselves were removed using the mmseqs taxonomy tool ‘–taxon-list '33,208&&!9913&&!9606'.

Protein coding sequences were predicted using TransDecoder (v5.7.1, ‘-m 75 –single_best_only’) (https://github.com/TransDecoder/TransDecoder), requiring a minimum length cut-off of 75 amino acids. Open reading frames (ORF) below the cut-off were included in case of significant homology scores identified using an MMseqs2 profile search against Pfam, eggNOG, or BLAST search against Swiss-Prot.

Finally, the contig with the longest, complete protein prediction from each De Bruijn graph component was chosen for subsequent analysis. In cases where only incomplete protein predictions were obtained, the contig with the longest predicted protein was chosen.

#### Phylotranscriptomics

The five transcriptomes generated in this study were supplemented with transcriptomes extracted from [20, 85] and performed gene supermatrix construction as described in [20]. Briefly, for each transcriptome, the longest protein per transcript was predicted using TransDecoder (v5.7.1, ‘-m 75 –single_best_only’), homologous groups were inferred using OrthoFinder [86] (v2.5.4, -M ‘msa’ -I 1.5 -z -ot -X), a multiple sequence alignment for each homologous group was created using MAFFT (v7.525) [67], and a gene tree was inferred using FastTree (v2.1.10) [87]. To arrive at a set of orthologous sequences for phylogenetic inference, the homologous groups were pruned using PhyloPyPruner (https://pypi.org/project/phylopypruner/) with midpoint rooting and ‘trim_lb 4, min_taxa = 25, Maximum inclusion, min_otu_occupancy = 0, min_gene_occupancy = 0’. The final supermatrix was composed of 1,701 protein alignments from 55 transcriptomes with 1,069,932 columns, 784,087 distinct patterns 552,857 parsimony-informative, 180,422 singleton sites, 336,653 constant sites and an overall percentage of missing data of 55.9%. The species tree was then inferred using IQ-TREE [69], first determining the best substitution model for each partition using ModelFinder (‘-m TEST’) and calculating 1,000 ultra-fast bootstraps and SH-like approximate likelihood ratio test with 1,000 replicates to assess branch support (-bnni -bb 1000 -alrt 1000).

## Results

### DNA barcoding analysis reveals taxon biases in planarian COI sequences available in GenBank

**Fig. 1:**
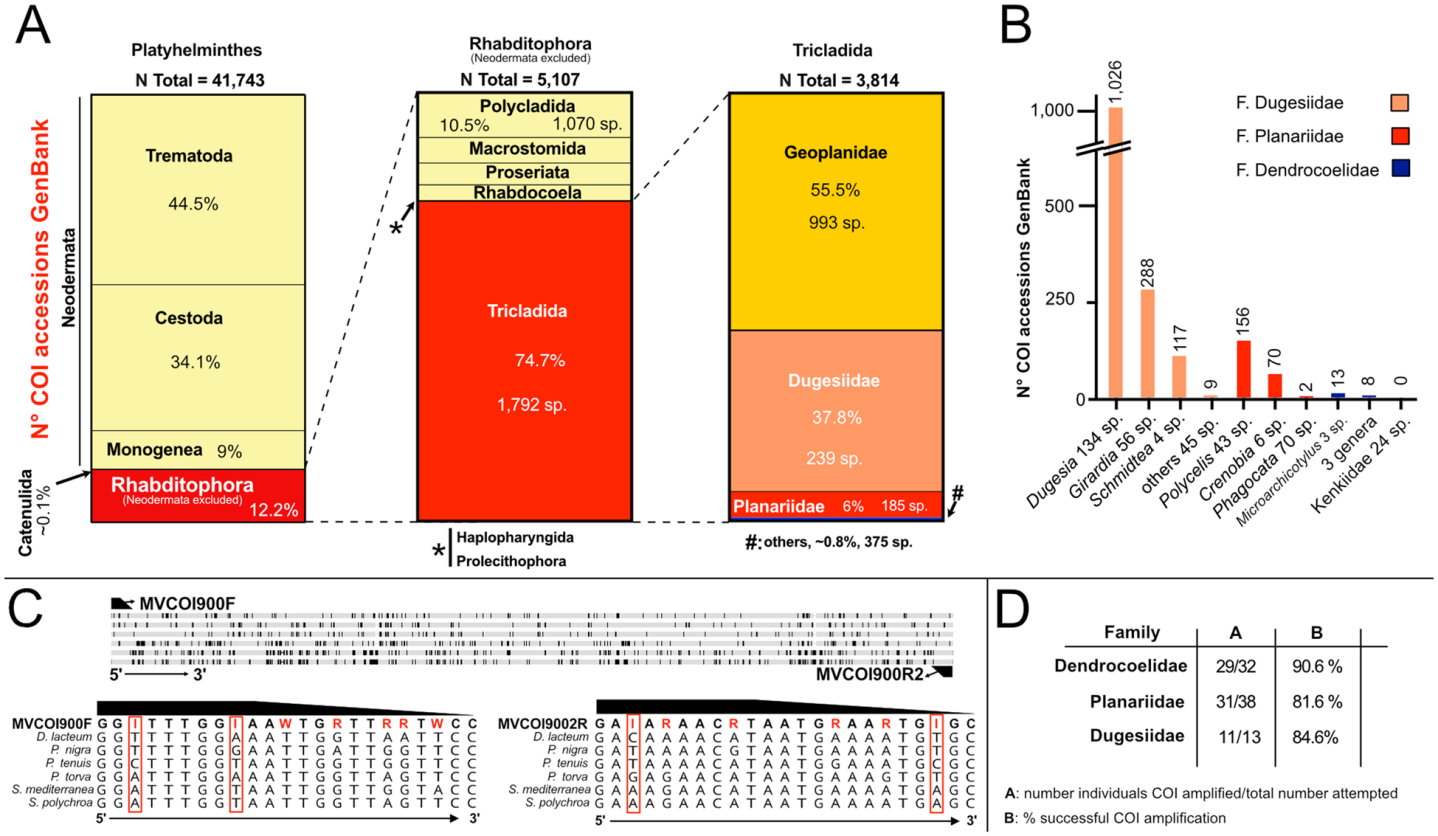
Underrepresentation of specific planarian groups amongst existing GenBank COI records. **A**. GenBank COI accession in three taxonomic groups: Platyhelminthes (left), Rhabditophora excluding Neodermata (middle), and Tricladida families (right). The total number of accessions (N Total), the percentage they represent from the total per group (%), and the approximate number of species (sp.) are indicated for relevant groups and subgroups. **B.** Number of GenBank COI accession for selected genera and approximate number of species (sp.). **C.** Degenerate primer design. Top, design of the COI primer pair MVCOI900 on basis of an alignment of the COI gene sequences from six planarian species. Primer binding positions are indicated. Black bars: variable sequence positions. Bottom, sequence alignment detail in the primer binding site. Primer highlighted in bold. Red: variable base positions in the primer. Red squares: highly variable positions represented by an inosine (I) base in the primer. For MVCOI900F, the first “W” wobble base in the primer sequence stems from an initially less stringent selection of sequences during primer design. **D.** Primer COI gene sequence amplification success by PCR

DNA barcoding holds great promise for streamlining integrative species description approaches [29]. To gauge its current specific utility for planarians, we first assessed the taxon coverage of COI reference sequences currently available in GenBank (National Center for Biotechnology Information, NCBI) [88]. Specifically, we compared the number of GenBank COI accessions for each major group within the Platyhelminthes to the number of species per group recorded in the “Turbellarian taxonomic database”, which we use as approximate estimates for comparative purposes [89]. Out of 41,743 platyhelminth COI entries, 87.6% were of parasitic Neodermata, which include medically relevant species like *Schistosoma mansoni* and, therefore, likely reflect a positive sampling bias (Fig. 1A). The remaining entries were split between Catenulida (0.1%) and the free-living Rhabditophora (excluding the parasitic Neodermata) (12.2%). Among those, Tricladida (planarians) comprised 74.7% of COI entries, a disproportionately high representation compared to other groups, such as Polycladida (10% of entries), considering the number of species (Fig. 1A). Within Tricladida, 55.5% of accessions belonged to Geoplanidae (land planarians, 55.4% of the known Triclad species). In comparison, freshwater Continenticola constituted 44.5% of the total number of accessions: 37.8% for Dugesiidae (13.1% of the known triclad species), 6% for Planariidae (10.3% of the known species), and only 0.6% for Dendrocoelidae (14.5% of the known species) (Fig. 1A). Notably, no COI sequences were available for Kenkiidae, a freshwater family with 24 described species, and only a single accession representing Cavernicola (nine species) was present in the data set. Finally, Maricola, a primarily marine triclad group with 83 known species, was represented by only eight accessions. Thus, the number of COI accessions is disproportionately high for the family Dugesiidae and disproportionately low for other freshwater groups like Planariidae or, particularly, Dendrocoelidae.

The genus *Dugesia* comprises 56.1% (134 sp.) of the known Dugesiidae species but accounts for 71.3% of the family accessions, indicating a notable overrepresentation. *Girardia*, which comprises 23.4% (56 sp.) of Dugesiidae species, constitutes 20% of accessions, while *Schmidtea*, with only four species, contributes disproportionately with 8.1% of accessions (Fig. 1B). In contrast, the remaining nine genera, collectively encompassing 13.8% of known species, account for only 0.6% of accessions (Fig. 1B). These findings reveal biases favouring *Dugesia* and *Schmidtea* within the Dugesiidae family. This trend of overrepresentation is mirrored in other planarian families. For example, within Planariidae, *Polycelis* and *Crenobia* are disproportionately well-represented, while several other genera are sparsely included in GenBank. These patterns within the currently available COI reference sequences suggest systemic biases in taxonomic sampling, which may become even more apparent with finer-scale analyses at the species level.

Overall, our results confirm the existence of pronounced taxonomic biases at both family and genus levels in current planarian barcode records in GenBank, which limits the utility of barcoding for planarian species identification.

### COI primer pair design for European freshwater planarians

One reason for the observed coverage biases in the COI GenBank record might be that the available primer pairs perform poorly on planariids (e.g. *Polycelis*) [90] and dendrocoelids (M. Riutort, M. Vila-Farré; unpublished observations). Towards the goal of improving primer performance in these clades, we designed a new primer pair (MVCOI900) that includes degenerate base positions or inosine as a universal base at highly polymorphic positions [91, 92] (Fig. 1C). MVCOI900 amplifies ~ 880 bp in the coding region of the COI gene that largely overlap with the amplicons generated by previous published primers (Fig. 1C; Additional files 1, 2). We tested the MVCOI900 primers' amplification efficiency on phenol–chloroform DNA preparations from a range of planarian species, as commercial DNA isolation kits perform poorly on planarians (see Methods). Amplification success rates with our standardised protocol were about 90.6% for dendrocoelids, 84.6% for dugesiids, and 81.6% for planariids (Fig. 1D). Within planariids, amplification was challenging for *Phagocata* or *Crenobia*, possibly indicating species- or genus-specific variability in primer efficiency and/or species-specific PCR inhibitors [93]. Analysis of additional *Crenobia* samples, though still showing cases of poor band amplification, provided a favourable assessment of the primer efficiency (about 33 of 37 samples yielded specific bands) (Additional fig. 3B). To evaluate the performance of MVCOI900 relative to available primer pairs, we performed a comparative analysis with the commonly used primer pair BarT/COIR, which produces an amplicon of similar size [66] (Additional files 2, 3). Interestingly, both primers achieved comparable amplification efficiencies, both under their respective standard conditions and with adjusted PCR conditions for difficult samples (Additional file 3). These results indicate that the initial assumption that published primers inherently perform poorly is not supported. Instead, the coverage biases in GenBank may reflect disproportionate focus on *Dugesia* and *Schmidtea* (to which the model species *D. japonica* and *S. mediterranea* belong), as well as earlier limitations in DNA extraction and PCR protocols that have since improved. In general, although BarT/COIR performs better than originally expected, neither primer pair is perfect. Consequently, the development of a truly universal COI primer for freshwater planarians remains an important objective. Nevertheless, our current approach yields broader taxonomic coverage than would be anticipated from the fragmented representation in GenBank, hence warranting the inclusion of barcoding in our integrative exploration of Croatian planarian diversity.

### Croatia field sampling campaign and preliminary classification of the planarian samples

**Fig. 2:**
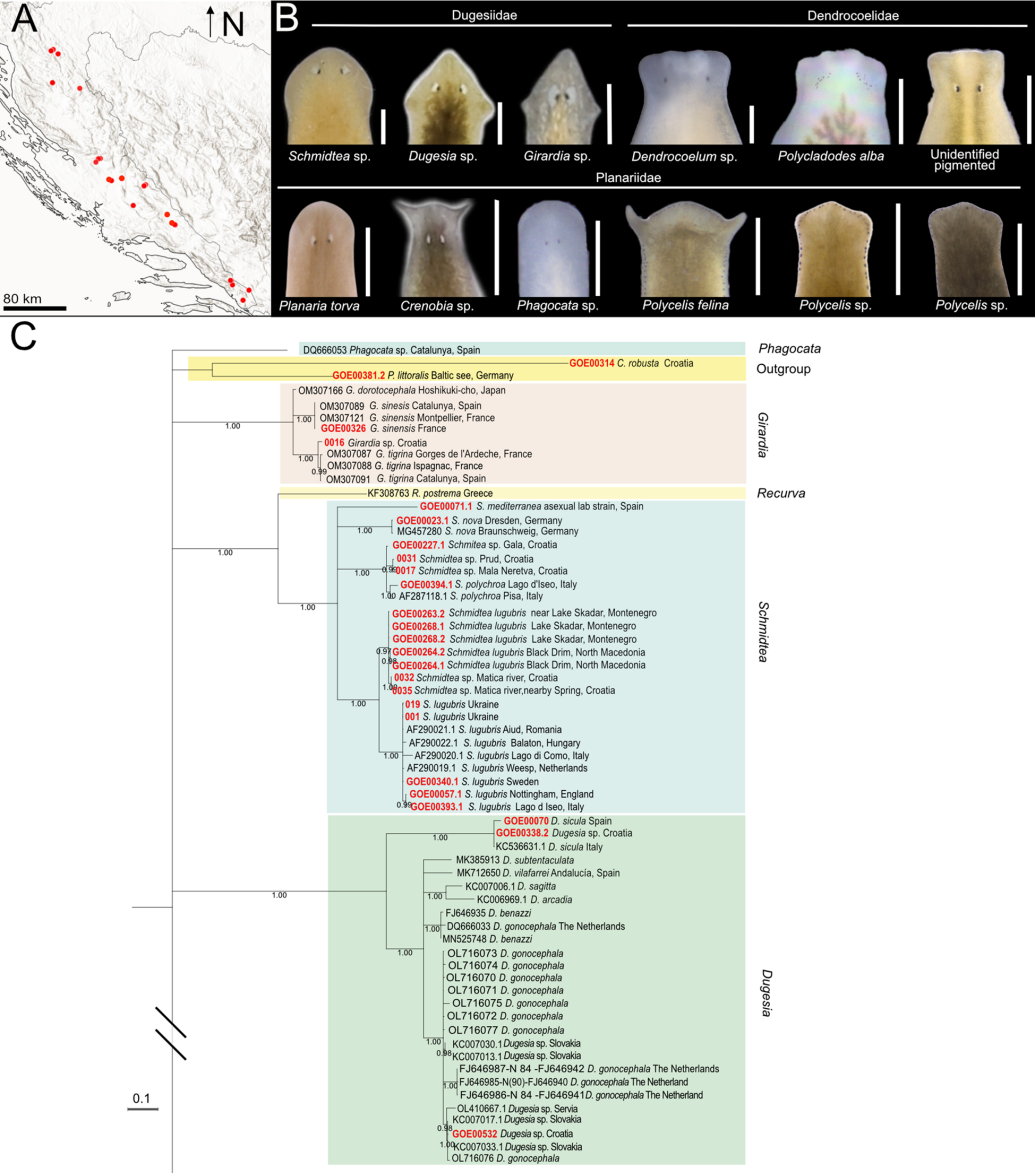

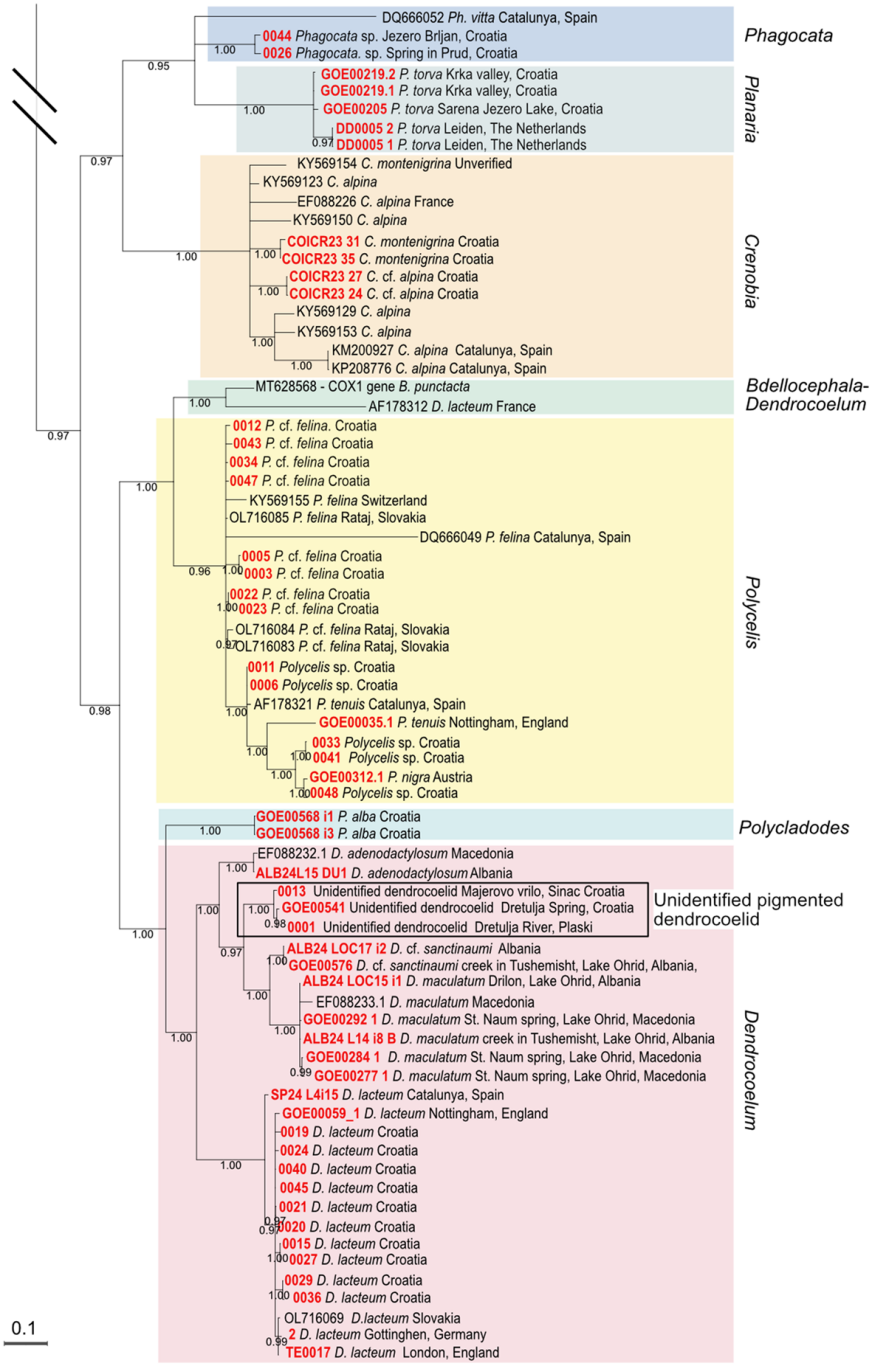
**a** Barcoding of the Croatian planarians, part 1. **A**. Field sampling sites with planarian occurrences. The sampling site at Ombla spring, north-east of Dubrovnik, is not indicated on the map. **B.** Preliminary taxonomic assignment of the collected specimens, based on morphological traits of head anatomy. Scale bar, 1 mm. **C.** First part of the maximum clade credibility (MCC) tree obtained from the sequence alignment *gap50* containing the indicated outgroups, dugesiids, and one *Phagocata* sample. The tree continues in b. Numbers at nodes represent posterior probability. Branches with posterior probability < 0.95 were collapsed for clarity. Red terminals correspond to individuals sequenced in this study. Colour boxes delimit planarian genera. b Barcoding of the Croatian planarians, part 2. Second part of the maximum clade credibility (MCC) tree obtained from the sequence alignment *gap50* containing the planariids and dendrocoelids, including the pigmented dendrocoelids collected in Croatia (See Fig. 2a for the first part). Numbers at nodes represent posterior probability. Branches with posterior probability < 0.95 were collapsed for clarity

To explore the diversity of the planarian fauna of Croatia, we sampled freshwater habitats in the country’s western karst region, including rivers, lakes, and springs (for sampling methodology, see [20] and Methods) (Fig. 2aA). The collected planarians were coarse-classified at the genus or species level based on external morphological features of live specimens (Fig. 2aB), guided by our taxonomic expertise and the existing literature on European and Western Balkan planarians [42–50, 94, 95].

The morphological characteristics used for genus identification included, among others, the number and arrangement of eyes relative to the body margin and head shape (Fig. 2aB illustrates the variability of this character), dorsal and ventral body colouration, number of visible pharynges, and shape and type of movement of the body margin. Our initial classification identified *Schmidtea* sp. (five localities), *Dugesia* sp. (two localities), and *Girardia* sp. (one locality) among dugesiids; *Dendrocoelum* sp. (11 localities), *Polycladodes alba* (one locality), and an unidentified pigmented dendrocoelid (three localities); and the planariids *Phagocata* sp. (three localities), *Planaria torva* (three localities), *Crenobia alpina* (one locality), *Crenobia montenigrina* (one locality), *Polycelis felina* (eight localities), and *Polycelis* sp., with auricles resembling those of *P. nigra* and *P. tenuis* (eight localities) (Fig. 2aB).

The unidentified pigmented dendrocoelid mentioned above was particularly interesting, as the only known pigmented dendrocoelid species in Europe outside the Ohrid and Prespa lakes region are *Bdellocephala punctata*, which possesses a different dorsal pigmentation [94], and the poorly known *Dendrocoelum lacteum verbanense* [96]. The Ohrid and Prespa lakes region is home to various pigmented *Dendrocoelum* species [97]. The collection of these remarkable specimens, approximately 600 km north of the Ohrid-Prespa region, highlights the scientific interest of Croatian triclads.

### DNA barcoding of Croatian planarians

To further characterise the collected specimens, we generated COI barcodes using the MVCOI900 primers for the Croatian samples and others collected during other European lab expeditions (78 sequences). Furthermore, we included existing GenBank COI sequences of European freshwater planarians (58 sequences) (Additional file 4), forming a dataset with 136 sequences (Dataset Barcoding). We combined those sequences into three alignments (*small*, *gap50* and *full* alignments) that varied in length and % of missing data, accounting for the variability in length introduced by the GenBank sequences (Additional file 2; Methods).

Notably, the MVCOI900 primers also amplified two maricolan planarians, *Procerodes littoralis* and Croatian individuals of *Camerata robusta*, which is only the second known population of this species [98], that we used as outgroups in the subsequent analyses. The expansion of planarian COI barcoding sequences, including poorly investigated planarian genera, e.g. *Dendrocoelum*, *Polycladodes*, European *Polycelis* or *Planaria*, represents a step forward in planarian barcoding and again highlights the utility of the new MVCOI900 primer set. All new sequences will be deposited in GenBank, with accession numbers provided in Additional file 4.

To assess the species identity of our samples, we generated phylogenetic trees with both Bayesian inference and Maximum likelihood (ML) (Fig. 2aC, 2b; Additional files 5, 6, 7, 8). We compared the similarity of our Croatian samples with the identified samples in the dataset. The traditionally recognised genera *Girardia*, *Dugesia* and *Crenobia* are recovered as monophyletic with statistical support in all analyses (Fig. 2aC, 2b; Additional files 5, 6). All *Planaria* samples cluster together. *Polycelis* and *Schmidtea* were supported in all cases except for the ML tree in the alignment *small* (Additional file 6), suggesting that this alignment lacks resolution. In contrast, *Dendrocoelum* and *Phagocata* are not supported in any analysis, with the barcodes *Dendrocoelum lacteum* (AF178312) and *Phagocata* (DQ666053) placed far from other members of their genera (Fig. 2a, 2b; Additional files 5, 6, 7).

Several Croatian samples were identified through matches in GenBank or our dataset. Among dugesiids, we identified *Girardia tigrina*, *Dugesia sicula*, *Schmidtea polychroa* and *Schmidtea lugubris* (Fig. 2aC, 3). We also identified the planariid *Planaria torva*, which clusters with previously confirmed *P. torva* barcodes (Fig. 2b). Intriguingly, *S. lugubris* consistently formed two sister clades in all Bayesian trees: one widespread across Europe and another restricted to the Adriatic region (from Croatia to Lake Ohrid) (Fig. 2aC; Additional file 5). The former clade was also supported in the ML trees (Additional file 6). This divergence warrants further investigation to clarify the taxonomic status of the two clades (see below).

**Fig. 3:**
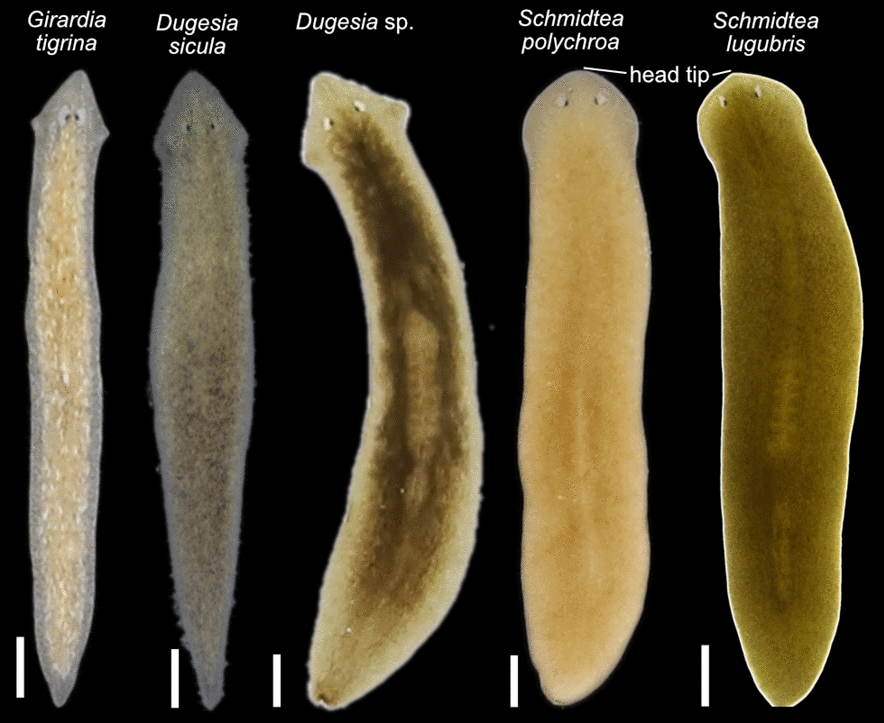
Diversity of dugesiids. Living planarian specimens representing the dugesiids collected during our expeditions. Scale bar, 1 mm

Barcoding also provided clues for unresolved samples. For instance, Croatian specimens initially assigned to *Dendrocoelum* sp. clustered with *D. lacteum* barcodes, except for the problematic barcode AF178312, originally labelled as *D. lacteum*. The Croatian sample GOE00532 (Fig. 2aC) grouped within a small clade containing three unidentified samples and a single *D. gonocephala*, suggesting it is likely *D. gonocephala* or a related form. Given this ambiguity, we considered it advisable to classify individuals from the same population using classical taxonomy. Notably, *D. gonocephala* DQ666033 [99] clustered with *Dugesia benazzi* in all analyses, indicating a likely misidentification (Fig. 2aC; Additional files 5, 6).

Due to the lack of resolution within the *Crenobia* clade, barcoding did not confirm our preliminary species assignment of Croatia specimens (Fig. 2b, Additional files 5, 6, 7). The single GenBank accession for *C. montenigrina* clustered separately from the putative Croatian *C. montenigrina*. Therefore, we will refer to the Croatian specimens assigned to *Crenobia alpina* as *Crenobia* cf. *alpina*. Specimens assigned to *C. montenigrina* were further investigated using classical taxonomic methods to clarify their species assignment (see below).

Barcoding was inconclusive for *Polycelis* samples, the two putative *Polycladodes alba*, two *Phagocata* sp., and three pigmented dendrocoelids (Fig. 2aC, 2b). All Croatian specimens with external morphology matching *Polycelis felina* formed a single clade with reference GenBank sequences of *P. felina* in both Bayesian and ML analyses for the *full* and *gap50* alignments, but with insufficient statistical support (PP = 0.90 and 0.92 in the two Bayesian trees; SH-aLRT = 85.9–88.6, UFBoot = 93–94 in the two ML trees) (see uncollapsed trees for the *Gap50* alignment in Additional file 7) (Fig. 2b). We therefore refer to the those specimens as *Polycelis* cf. *felina* pending further study.

The *Polycladodes alba* and Croatian *Phagocata* sequences had no closely related matches in GenBank. Meanwhile, the three pigmented dendrocoelids clustered with members of the genus *Dendrocoelum*, including pigmented species from Lake Ohrid, suggesting they likely belong to this genus. Interestingly, those three individuals (each one from a different locality) are grouped by geographic proximity into two clusters (Fig. 2b).

Overall, this analysis again highlights the fragmentary record of COI accessions in GenBank for many widespread European taxa (e.g. *P. torva*, several *Polycelis* species, *D. lacteum*, or *P. alba*) and the difficulties that result from barcoding-based species identification (Fig. 3).

### Phylogenetic relationships of the Croatian dendrocoelids and transcriptomic comparison of the *Schmidtea lugubris* clades

**Fig. 4:**
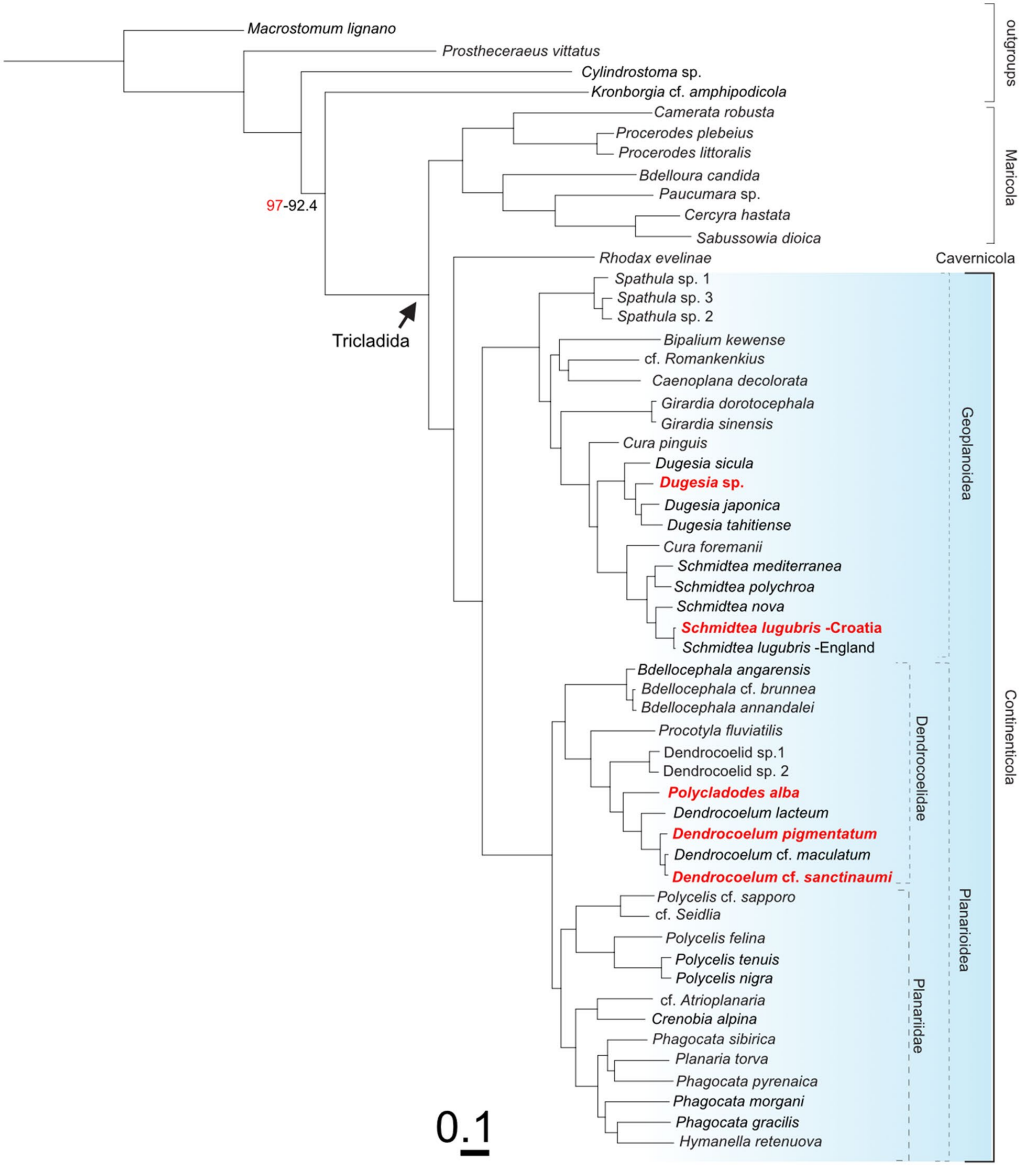
Phylogenetic relations of Croatian dendrocoelids and *Schmidtea lugubris.* Transcriptome-based ML tree with transcriptomes de novo generated for this study highlighted in red. Colour shading marks the Continenticola, which contains the freshwater groups analysed. SH-aLRT and UFboot Bootstrap values 100, except where indicated (SH-aLRT in red; UFboot Bootstrap in black)

To obtain further insights into the phylogeny of Croatian dendrocoelids and the two *Schmidtea lugubris* clades revealed by our barcoding effort, we generated de novo assembled transcriptomes via our pipeline [59, 60] of *Polycladodes alba*, the pigmented dendrocoelid, *Dendrocoelum* cf. *sanctinaumi* (a pigmented form from the Ohrid region), *Dugesia* sp. (that we plan to include in future studies), and a Croatian population of *S. lugubris*. Together with the recently published transcriptomes of four *Schmidtea* species [85] and the transcriptomes published in [20], this amounted to a dataset of 55 transcriptomes (51 planarians and four outgroups). Of note is that two transcriptomes in the tree also correspond to Croatian population of *S. polychroa* and *P. torva,* which we published previously [20, 85]. We extracted broadly conserved orthologues [69, 84, 86] and constructed a phylogenetic tree (see Methods). The resulting phylogeny achieved maximum branch support for all but one node (Fig. 4). Consistency between our phylogeny and that in [20] (species common to both trees appear in a similar position, forming similar phylogenetic clades) underscores the robustness of the tree topology and validates the inclusion of new transcriptomic data.

The taxonomic status of *Polycladodes* has been debated, with some earlier publications considering it a subgenus of *Dendrocoelum* [46]. We adopt the use of the genus name *Polycladodes* Steinmann, 1910, over *Dendrocoelum* Örsted, 1844, for *P. alba*, following recent taxonomic classifications [100, 101]. Our analysis positions *Polycladodes alba* as a sister group to the four *Dendrocoelum* species included in the phylogeny, which is compatible with this option (Fig. 4).

In our phylogenetic tree, the Croatian pigmented dendrocoelid forms a clade with two other pigmented species from Ohrid, supported by maximum branch support and with relatively shallow branch lengths, indicating a close relation among the species (Fig. 4). This result suggests that it is a member of the genus *Dendrocoelum*, in agreement with the barcoding data, and highlights the possibility of a shared evolutionary history of pigmented *Dendrocoelum* species in the region.

To understand whether the two clades of *S. lugubris* (Fig. 2aC) are different enough to constitute separate species, we compared the branch length distance between the sister species of *Schmidtea* and the two *S. lugubris* forms*.* Our tree confirms the established relationships within *Schmidtea* [85, 102] (Fig. 4): *S. mediterranea* is sister to *S. polychroa,* and *S. lugubris* is sister to *S. nova*. The branch length distances between *S. mediterranea* and *S. polychroa* (0.1104122901) and between *S. nova* and *S. lugubris* (British population) (0.1161586304) are comparable. In contrast, the distance between the two *S. lugubris* clades (0.0109926202) is approximately ten times smaller, suggesting a much lower divergence. This lower divergence in the phylogenetic tree, together with the differentiation into clades in the COI barcoding analysis (Fig. 2aC), points to the existence of previously unknown intraspecific variability and genetic structure rather than the split into two species of *S. lugubris*.

**Fig. 5:**
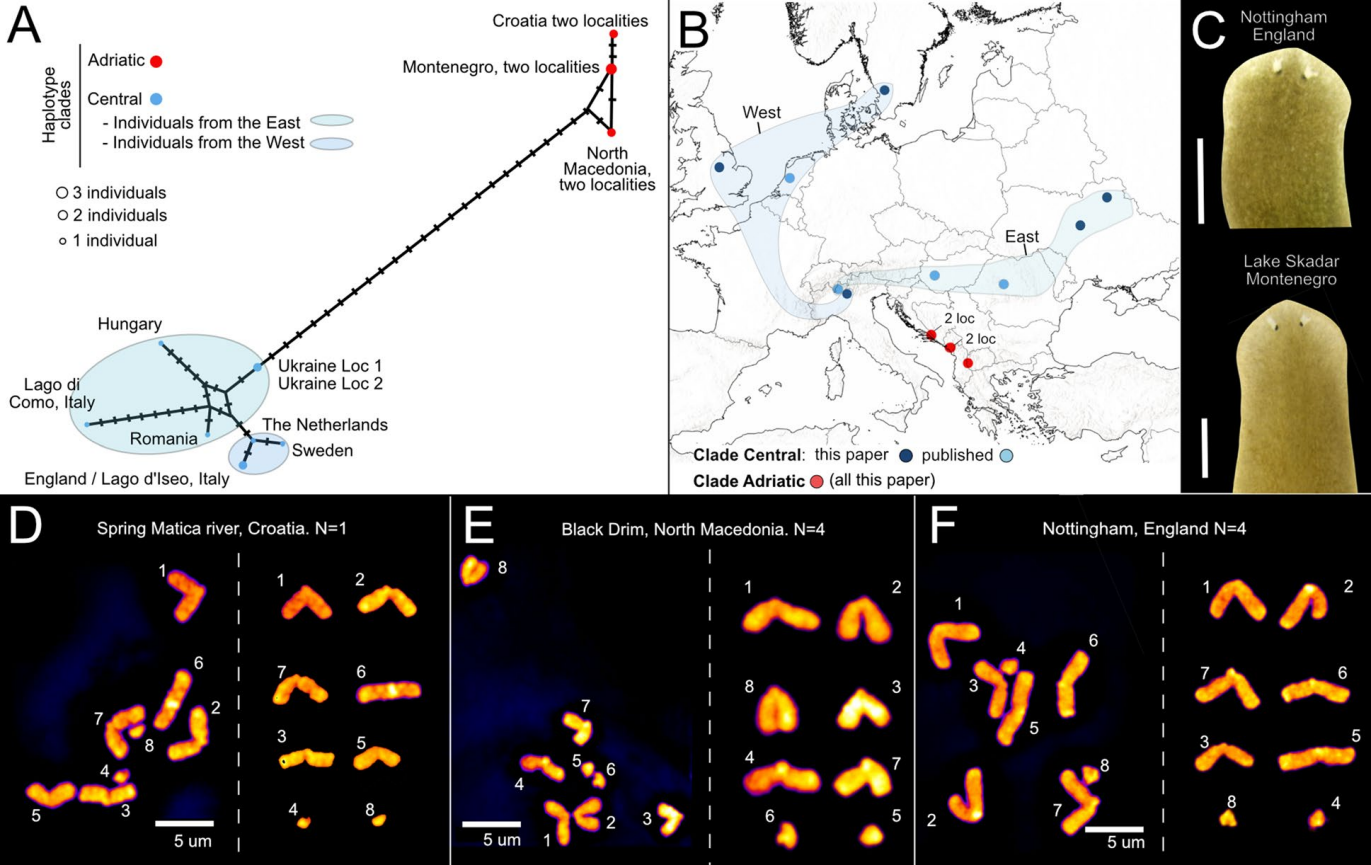
The Adriatic populations of *Schmidtea lugubris* form a distinct genetic clade. **A**. Haplotype network for the mitochondrial gene COI from Dataset 1 (sequence length 308 bp). Each circle represents a different haplotype, and the size of the circle indicates the frequency of each haplotype. Black bars represent intermediate (non-present) haplotypes, and lines connecting haplotypes (existing or not) represent one nucleotide change. The colour scheme is used to differentiate the major haplotype clades (see legend) and the origin of the individuals sampled within the central clade. **B.** Distribution map of the two haplotype clades. Each dot represents a locality. Dots representing two nearby localities are indicated (2 loc). Curves enveloping localities correspond to the Eastern and Western localities described in **A** for the central clade. The origin of COI sequences used to assign each locality to a clade (published data or this study) is indicated. **C.** Living specimens showing the characteristic pointed head of *Schmidtea lugubris.* **D-F.** Metaphasic plate (left) and chromosome complements arranged in pairs (right) of individuals from three populations. Numbers indicate the correspondence between chromosomes. Individuals from the three populations show diploid karyotypes (2n = 8) with three acrocentric and one small chromosome. The number of individuals analysed per population (N) is indicated next to the population name

We constructed two COI datasets with sequences of different lengths to investigate the genetic diversity of the two *S. lugubris* clades, Dataset 1 (308 bp) and 2 (702 bp) (see Methods). We identified 10 haplotypes in Dataset 1 and 8 in Dataset 2. The haplotype networks separate two well-differentiated groups (Fig. 5A, Additional file 9) that we named the Central and Adriatic clades. In Dataset 1, the Central clade encompasses individuals distributed across central Europe, with minimal divergence across a large area (from the Netherlands to Sweden, the United Kingdom, and northern Italy), as well as populations in the eastern regions of Europe forming a more complex network (Italy, Hungary, Romania, and Ukraine) (Fig. 5A, B). Individuals in the east of the Central clade exhibit no shared haplotypes with the Western individuals or among them. The Adriatic clade consists of three haplotypes in Dataset 1, restricted to Croatia, Montenegro, and North Macedonia. The Adriatic clade displays minor genetic differentiation between the sampled individuals in this dataset.

Conversely, in Dataset 2, the Adriatic clade gains haplotype diversity (from three to four haplotypes), and the three areas of origin are better differentiated, indicating the existence of interesting diversity in the Adriatic zone as well. Long sequences from the Central clade are scarce, resulting in only four haplotypes in Dataset 2. Notably, the Ukrainian individuals remain distinct from the Western individuals. In summary, our analysis consistently revealed the existence of two highly distinct genetic clades within *S. lugubris*.

### Systematic section

To address some of the ambiguities in our barcoding analysis, we conducted a detailed histological analysis of individuals from populations where enough material was available, a standard method in triclad taxonomy [26, 50, 97]. *Planaria torva* was included to curate the submitted COI accession as the first GenBank COI accession of this species. The systematic section as a whole presents data for five taxa in three families: the dugesiid *Schmidtea lugubris*, the dendrocoelids *Polycladodes alba* and the pigmented dendrocoelid (described as a new species, *Dendrocoelum pigmentatum*), and the planariids *Planaria torva* and *Crenobia montenigrina*.

Family: Dugesiidae Ball, 1974

Genus: ***Schmidtea*** Ball, 1974

Species: ***Schmidtea lugubris*** (Schmidt, 1861)

**Material examined**. H0273, Matica river, nearby Spring, Dusina, Croatia, (43.1762° N, 17.4069° E), 15 September 2021, coll. Jochen C. Rink, Miquel Vila-Farré, Ludwik Gąsiorowski, Uri Weill and Rick Kluiver, sagittal sections on 20 slides; H0274, ibid., horizontal sections on 10 slides; H0275, ibid., sagittal sections on 10 slides; H0802, laboratory population originally collected from Nottingham, United Kingdom (52.942432° N, −1.113739° E), December 2011, coll. Jochen C. Rink, sagittal sections on 12 slides; H0803, ibid., sagittal sections on 8 slides; H0804, ibid., sagittal sections on 7 slides; H0805, laboratory population originally collected from Lago d’Iseo, Italy (45.724999° N, 10.053001° E), May 2013, coll. Miquel Vila-Farré, sagittal sections on 9 slides; H0806, ibid., sagittal sections on 7 slides; H0807, ibid., sagittal sections on 7 slides; H0813, laboratory population originally collected from Lake Skadar, Karuč, Montenegro (42.35791° N, 19.10661° E), September 2014, col. Jochen C. Rink, Miquel Vila-Farré, Helena Bilandžija, sagittal sections on 11 slides; H0814, ibid., sagittal sections on 11 slides; H0815, ibid., sagittal sections on 9 slides; H0816, laboratory population originally collected from Crni Drim (Black Drim, Ohrid outflow), Republic of North Macedonia (41.355435° E, 20.623436° N), September 2014, col. Jochen C. Rink, Miquel Vila-Farré, Helena Bilandžija, sagittal sections on 23 slides; H0817, ibid., sagittal sections on 15 slides; H0818, ibid., sagittal sections on 11 slides.

### Taxonomic discussion

Wild Croatian *S. lugubris* individuals measure up to ~ 1.2 cm in length and present a uniformly brown dorsal pigmentation. Other strains of *S. lugubris* maintained in our collection [20] are mostly brown. The characteristic but challenging-to-detect pointed head of *S. lugubris* [103], which contrasts with the rounded heads of *S. polychroa*, is visible in some individuals from both the Central and the Adriatic clade (Fig. 3, 5C). The copulatory apparatus anatomy conforms with that expected for *S. lugubris*. They present two seminal vesicles, the first narrow in the middle and broader in its upper and lower sections and coated with a well-developed layer of circular and longitudinal muscles. The narrow ejaculatory duct emerges from an area close to the ventral section of the seminal vesicle. It later widens to form the second vesicle, which is smaller, providing a strong muscle layer that combines longitudinal and circular muscles. Once in the very large penis papilla, the ejaculatory duct runs initially centrally to bend markedly in the middle section of the papilla to run again centrally to open into a very long and distinctive nipple characteristic of the species (Additional file 10). Of note, several individuals from the United Kingdom and one from North Macedonia (H0802, H0803, H0804 and H0818) present an expansion in one of their vasa deferentia. The copulatory apparatus of *S. lugubris* in individuals of other localities is similar: the penis bulb and the two seminal vesicles are surrounded by very muscular tissue, the ejaculatory duct follows a similar trajectory, the penis papilla is large and presents a nipple that, although variable in length, is present in all the individuals analysed (Additional files 10, 11).

### Karyological analysis

Karyological studies clarified the complex taxonomic history of the four *Schmidtea* species by identifying seven chromosomal strains (biotypes) (see [102] for a summary). *Schmidtea lugubris* corresponds to biotype E, diploid with a karyotype 2n = 8 and three acrocentric (terminal or nearly terminal centromeres) and one submetacentric chromosome [102, 104]. Our analysis revealed similar karyotypes in Adriatic and Central clade individuals. All metaphase plates analysed (a total of 54; see methods) showed eight chromosomes, except for five plates, which exhibited 7, 9, or 12 chromosomes. Among the eight chromosomes, six were acrocentric, while the small size of the remaining two chromosomes precluded counting their arms. This karyotype 2n = 8, with a haploid complement of three large acrocentric and one small chromosome (Fig. 5D-F), aligns with biotype E, confirming the specimens as *S. lugubris*. Thus, our analysis, integrating a multigene phylogeny, barcoding, anatomical, and karyological data, strongly suggests that the Adriatic and Central clades correspond to distinct genetic lineages, albeit with similar anatomy and karyology, thus representing intraspecific variation within *S. lugubris*.

Family: Dendrocoelidae Hallez, 1892

Genus: ***Polycladodes*** Steinmann, 1910

Species: ***Polycladodes alba*** Steinmann, 1910

**Material examined.** H0218, Dretulja Spring, Plaški, Croatia (45.0745° N, 15.3428° E), 13 September 2021, coll. Jochen C. Rink, Miquel Vila-Farré, Ludwik Gąsiorowski, Uri Weill and Rick Kluiver, sagittal sections on 20 slides; H0219, Ibid., sagittal sections on 11 slides; H0658, Dretulja Spring, Plaški, Croatia (45.0745° N, 15.3428° E), 19 June 2023, coll. Miquel Vila-Farré, sagittal sections on 18 slides.

### Taxonomic discussion

**Fig. 6:**
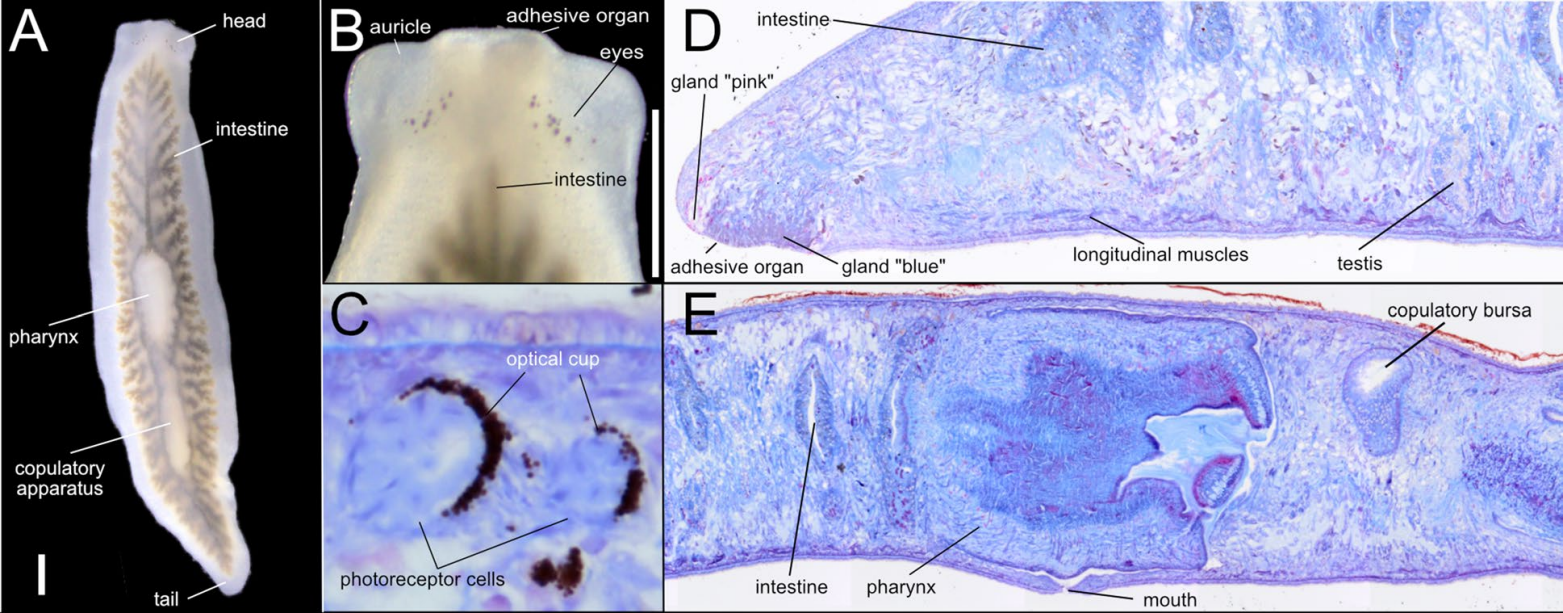
External and internal anatomy of *Polycladodes alba* from Croatia. **A-B.** Live specimens. Scale bar, 1 mm. **A**. Intestinal morphology, revealed by the ingested food.** B**. Detail of the head. **C-D.** Brightfield image of sagittal sections H0218. **C**. Anatomical details of two eyes. **D.** Anterior section of the body showing the adhesive organ. **E.** Middle section of the body showing the central position of the mouth in the pharyngeal pouch

**Fig. 7:**
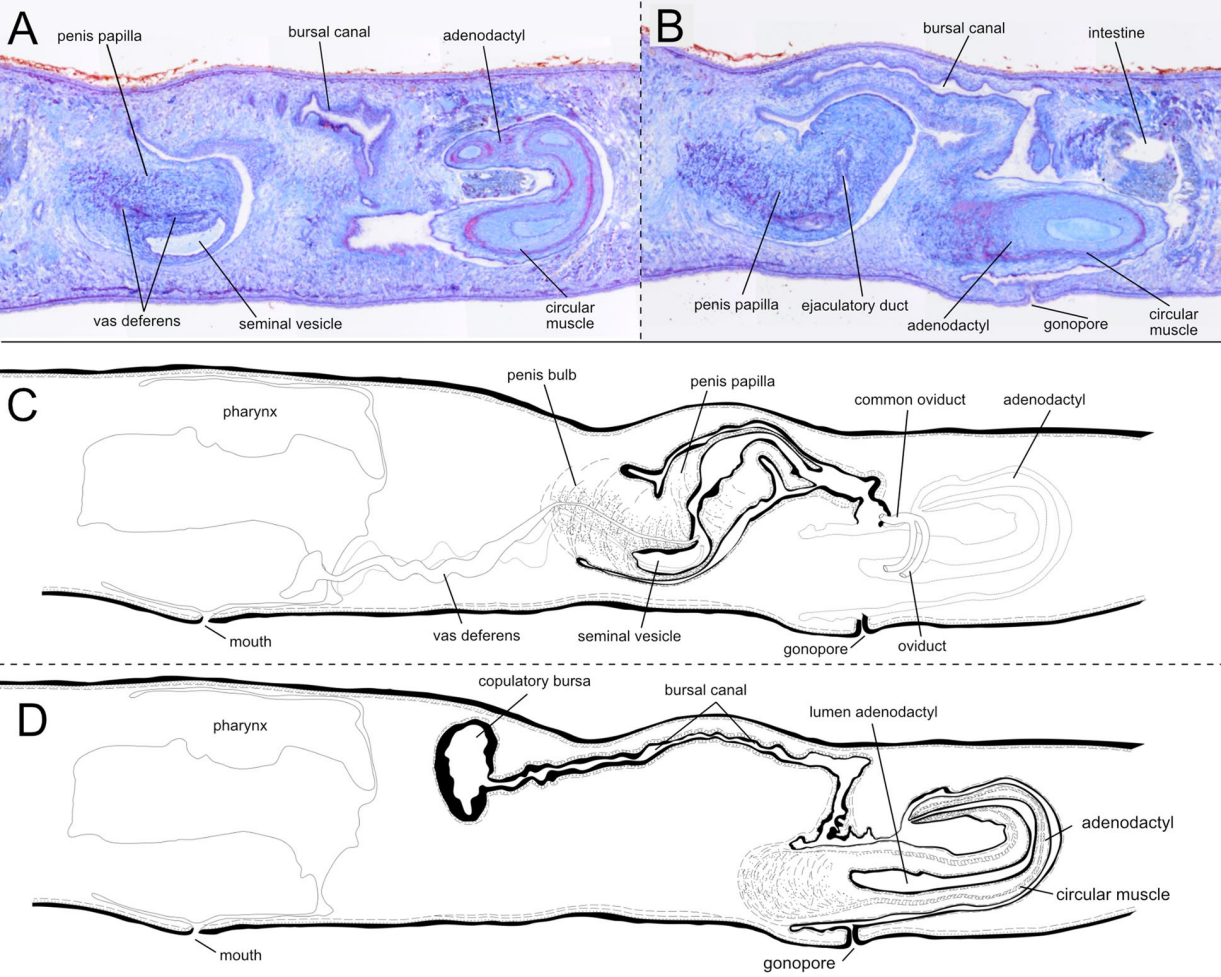
The copulatory apparatus of *Polycladodes alba.* **A-B.** Brightfield image of sagittal sections. H0218. **A**. Detail of the base of the penis papilla and the distal section of the adenodactyl. **B.** Detail of the penis papilla, bursal canal and base of the adenodactyl. **C-D.** Diagrammatic reconstruction of the copulatory apparatus. **C.** Section containing the long penis papilla. **D.** Section containing the copulatory bursa, bursal canal, adenodactyl, and gonopore

The most distinctive external anatomical feature of *P. alba* is the presence of two separate fields of eyes and its unpigmented colouration (Fig. 2B and Fig. 6A-C). *Polycladodes alba* is further characterised by an anterior intestinal branch that does not reach the level of the eyes (Fig. 6B), a well-developed anterior adhesive organ (Fig. 6B, D), a short pharynx with the mouth located approximately in the middle of the pharyngeal pouch [105] (Fig. 6E), in the external pharynx epithelium the circular muscle layer is entally followed by a layer of longitudinal muscles [46], predominantly ventral testes (Fig. 6D), a very long penis papilla (Fig. 7A-C), vasa deferentia that open into the anterior section of the seminal vesicle inside the penis papilla (Fig. 7C), and a very long adenodactyl with a very long free papilla (Fig. 7A-B, D).

In the Croatian specimens, the testes are predominantly ventral, although some are dorsally located, as observed in individuals H0218 and H0658. The copulatory bursa is small in the two analysed specimens (H0218, H0219). The bursal canal is long, consistent with individuals from other populations [106]. Interestingly, the adhesive organ in the Croatian specimens contains glands. However, the organ is less developed than that described by de Beauchamp [46], appearing less conical and lacking the "suction cup" shape (Fig. 6D). Of note, the Croatian specimens present in the adenodactyl a circular muscle band with longitudinal muscles ectally and entally distributed to the circular muscle (Fig. 7B, D). An adenodactyl with a muscular structure reminiscent of the so-called Balkan type of adenodactyl is present in several species of *Dendrocoelum*, broadly distributed in Europe, including the Western Balkans [107].

Genus: ***Dendrocoelum*** Örsted, 1844

Species: ***Dendrocoelum pigmentatum*** Vila-Farré, sp. nov.

**Material examined**. Holotype: H0366, Majerovo vrilo, Sinac, Croatia (44.8147° N, 15.3579° E), 13 September 2021, coll. Jochen C. Rink, Miquel Vila-Farré, Ludwik Gąsiorowski, Uri Weill and Rick Kluiver, sagittal sections on 8 slides. Paratypes: H0364, ibid., sagittal sections on 11 slides; H0222, ibid., sagittal sections on 13 slides; H0220, ibid., horizontal sections on 9 slides. Other material: H0293, Dretulja River, Plaški, Croatia (45.085° N, 15.3663° E), 13 September 2021, coll. Jochen C. Rink, Miquel Vila-Farré, Ludwik Gąsiorowski, Uri Weill, and Rick Kluiver, sagittal sections on 16 slides; H0294, ibid. sagittal sections on 33 slides; H0296, Dretulja Spring, Croatia (45.0745° N, 15.3428° E), 13 September 2021, coll. Jochen C. Rink, Miquel Vila-Farré, Ludwik Gąsiorowski, Uri Weill and Rick Kluiver, sagittal sections on 14 slides; H0297, ibid. sagittal sections on 13 slides. One individual per locality was barcoded; corresponding GenBank accession numbers will be uploaded to GenBank.

### Diagnosis

With respect to external features, *Dendrocoelum pigmentatum* can be distinguished from its congeners by the presence of dorsal pigmentation in the adult, including the head, and a pale cross-like pattern. Anatomically, it differs from pigmented congeners by a moderately developed adhesive organ, strong subepidermal musculature, a penis papilla with strong circular musculature that may be partially invaginated, vasa deferentia opening into the lower part of the seminal vesicle, a bursal canal that narrows before opening in the atrium, and an adenodactyl positioned ventral and lateral to the penis, similar to the penis in length, and shorter than the pharynx.

### Etymology

The epithet is derived from the Latin adjective *pigmentatum*, meaning pigmented, painted, or coloured. It alludes to the presence of body pigmentation in the species.

### Habitat

**Fig. 8:**
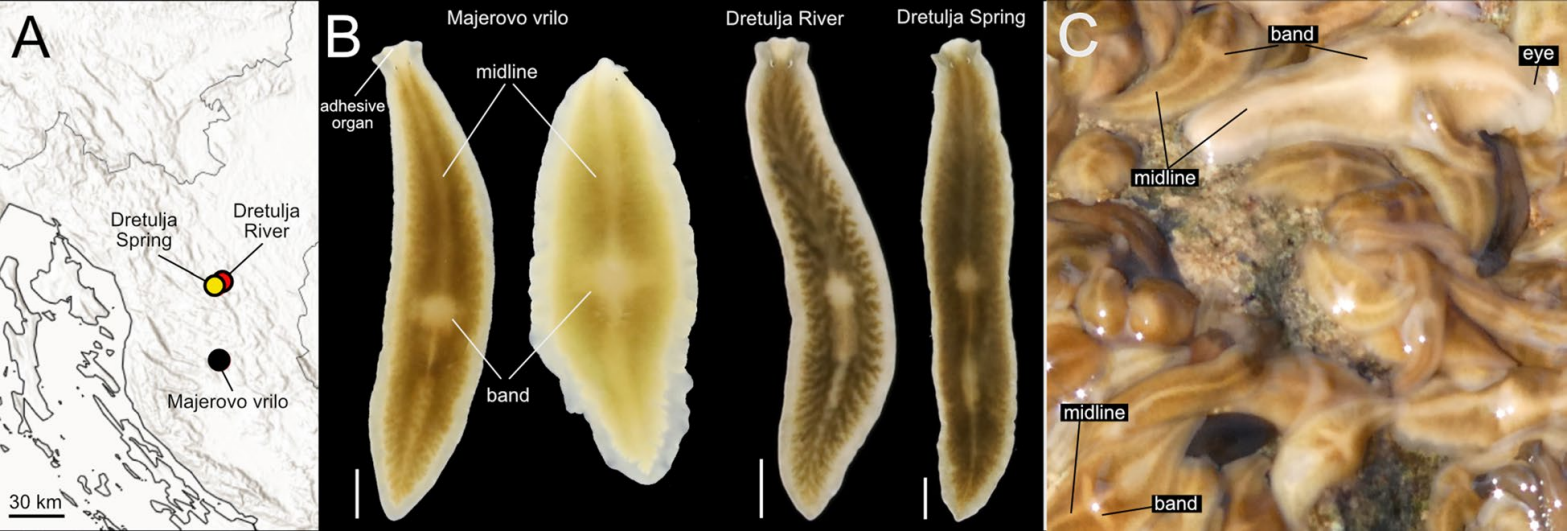
Distribution and pigmentation pattern of *Dendrocoelum pigmentatum*. **A**. Distribution of three known populations. **B**. Living animals from the three populations, imaged under laboratory conditions. Animals from Majerovo vrilo were imaged together to show the intrapopulation variability in pigmentation. Scale bar, 2 mm. **C.** Live image of animals in Majerovo vrilo in their habitat. The striking cross-like design formed by the dorsal midline and transverse bands is evident

*Dendrocoelum pigmentatum* has been collected from three nearby localities in the Croatian karst (Fig. 8A). One population occurs at Majerovo vrilo (visited by us twice), one of the three major springs of the Gacka River [108], approximately 30 km from the other two and separated by the Mala Kapela mountain range. The Gacka flows 11 km before sinking underground for ~ 20 km, re-emerging to ultimately discharge into the Adriatic Sea [109].

The other two populations inhabit the Dretulja River system: one at its source, Dretulja Spring (visited twice), and the other about 2.2 km downstream near Plaški (visited once). The Dretulja is a tributary of the Mrežnica [110], which belongs to the Danube system and ultimately flows into the Black Sea. Despite their proximity, the Majerovo vrilo and Dretulja populations are situated in distinct drainage basins—Adriatic and Black Sea—highlighting potential biogeographic and ecological differentiation.

At Majerovo vrilo (Sinac), the species is abundant in the outlet immediately below the mill. This habitat has a depth of less than 50 cm, a stony bottom, and is interspersed with aquatic vegetation. Here, *D. pigmentatum* co-occurs with *Polycelis* cf. *felina*, *Polycelis* sp., *Phagocata* sp. and an unidentified white dendrocoelid.

At the outlet of the Dretulja Spring, approximately 7 m wide, the habitat features a stony substrate and abundant aquatic vegetation. In this locality, *D. pigmentatum* is relatively common and co-occurs with *Polycladodes alba* (rare) and *Polycelis* cf. *felina* (abundant).

Finally, at the Dretulja River near Plaški, the river measures approximately 10 m in width and has a depth of around 80 cm. This site is situated upstream of a fish farm. Here, *D. pigmentatum* was less common during our single visit to the locality and coexisted with *Polycelis* cf. *felina*. In the three localities where it occurs, *D. pigmentatum* is found in flowing water habitats.

### Description

Live specimens with a body length of up to approximately 25 mm and a width of ~ 4 mm in the central part of the body and approximately 2.5 mm at the level of the eyes The distance of the two eyes from each other is about one-third of the transverse diameter of the head, and their approximate distance from the frontal margin is longer than that from the lateral margins. There is a constriction at the level of the eyes, which are situated apart (Fig. 8B). Anterior end truncated and provided with a pair of lobulated latero-anterior projections, and a median lobe (Fig. 8B). Live animals are pigmented with brown dorsal colouration. In Majerovo vrilo, the degree of pigmentation varies substantially between individuals, with some being much paler than the rest (Fig. 8B, C). In the Dretulja River populations, the observed individuals have a similar brown dorsal colouration (Fig. 8B). The adhesive organ zone has a more creamy pigmentation that forms a triangular area extending backwards to the level of the eyes. A rim in the body margin is lighter or almost unpigmented. There is a pale midline along the anteroposterior axis of the animal, starting behind the eyes and reaching almost to the tail. A pale transversal band extends over the region of the pharynx, reaching laterally about halfway between the mid-line and the lateral rim. The pale transversal band and the dorsal midline form a cross-like pattern very conspicuous under field conditions (Fig. 8C). In animals imaged under laboratory conditions, this “cross” can resemble a “dot” (Fig. 8B).

**Fig. 9:**
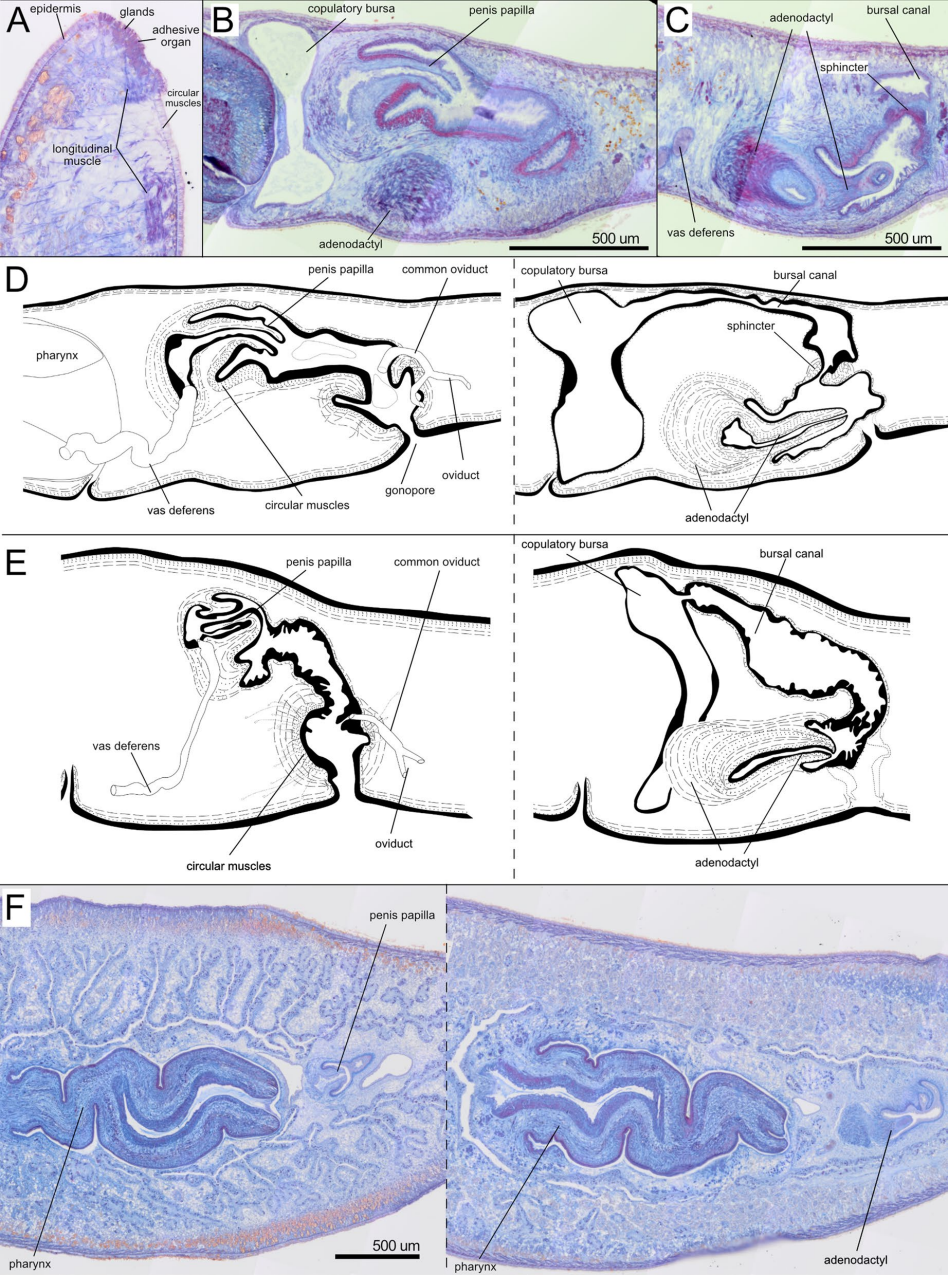
Internal anatomy of *Dendrocoelum pigmentatum*. **A** H0222. Brightfield image of sagittal sections. Detail of the adhesive organ**. B-D** H0366 **B-C.** Brightfield image of sagittal sections. **B**. Detail of the penis papilla and the atrium. **C.** Detail of the adenodactyl. **D.** Diagrammatic reconstruction of the copulatory apparatus. Left, section containing the penis papilla, male atrium and gonopore. Right, section containing the adenodactyl, copulatory bursa and the bursal canal. **E.** H0293. Diagrammatic reconstruction of the copulatory apparatus. Left, section containing the penis papilla, male atrium and gonopore. Right, section containing the adenodactyl, copulatory bursa and the bursal canal. **F.** Brightfield image of horizontal sections H0220. Detail of the pharynx and the copulatory apparatus

The subterminal anterior adhesive organ is only moderately developed, consisting of a shallow cup made up of epithelial cells, which are pierced by numerous openings of glands (Fig. 9A). The very thick anterior ventral longitudinal body musculature is interrupted at the level of the adhesive organ, in a parenchymal area almost devoid of longitudinal fibres in H0222. Right after, a few longitudinal fibres at the same level as the ventral longitudinal muscles are associated with the adhesive organ (Fig. 9A).

The pharynx is located approximately in the middle of the body and measures less than 1/4th of the body length in specimens from Majerovo vrilo (H0222, H0364 and H0366), while it is longer than 1/4th of the body length in specimens from the Dretulja River H0293, H0296. The mouth is situated at the posterior end of the pharyngeal pocket in specimens from Majerovo vrilo (H0222, H0364 and H0366) but slightly shifted anteriad for some distance in histological sections of individuals from populations along the Dretulja River (H0293, H0294, H0296 and H0297).

The two ovaries occur on the ventral side of the anterior region, behind the brain, in individuals H0364 and H0366. The oviducts originate from the laterodorsal part of the ovaries and are provided with a slight expansion at their anterior end, the tuba. The oviducts run posteriorly, converging behind the copulatory apparatus to form a single common oviduct (Fig. 9D-E). The common oviduct runs anteriorly and opens into the distal end of the male atrium before the latter connects with the common atrium. The distal part of the oviducts and the first tract of the common oviduct receive the openings of the shell glands in individuals H0366 and H0294.

The numerous well-developed testes are mostly ventral, but can extend to the dorsal surface of the body and from behind the ovaries to close to the posterior end of the body. In specimen H0366, the sperm ducts form very large spermiducal vesicles, packed with sperm, between the last section of the pharyngeal pouch and the mouth. The copulatory apparatus is localised immediately behind the pharyngeal pocket. In specimens H0366 and H0293, the copulatory bursa has the shape of a large sack that occupies almost the entire dorso-ventral diameter of the body (Fig. 9B, D, E). The bursa is lined with a thin epithelium that is taller at the level of the penis bulb. In H0366, the bursal canal runs posteriorly to the side of the penis and widens at its distal part, forming an enlargement at the end, then turns ventrally and narrows before opening into the common atrium. In other specimens, the bursal canal widens towards the centre before narrowing when opening on the common atrium (H0364, H0293). The meeting point between the atrium and the bursal canal is highly muscular and resembles a sphincter in individuals from Majerovo vrilo (H0222, H0364 or H0366) (Fig. 9C; Additional file 12), but not in individuals of other populations.

The penis bulb and the penis papilla are positioned dorsally and laterally to the adenodactyl (Fig. 9D, E, F). The muscular penis bulb, formed by longitudinal and circular muscle fibres, is of moderate size. The vasa deferentia penetrate the penis bulb separately at its middle part. Then, they open separately into the lower section of the long seminal vesicle, which is housed by the bulb. The seminal vesicle communicates with the ejaculatory duct that opens at the tip of the penis papilla. The penis papilla is covered by an epithelium that at the basal part is thinner than at the distal part. The epithelium is underlain by a thick layer of circular muscle in the proximal section of the penis papilla, particularly on the ventral side. This layer becomes thinner distally and is absent at the papillary tip. This thick muscle layer extends in the ventral section of the atrium close to the base of the penis papilla (Fig. 9B, D). In some specimens, e.g. H0293 and H0222, the penis papilla appears partly invaginated into the large papilla lumen (Fig. 9E). The length of the extended penis papilla is similar to that of the papilla of the adenodactyl in H0366 (Fig. 9D), and longer in H0296. The penis papilla projects into a long and wide male atrium, which is lined by a nucleated epithelium and surrounded by a subepithelial layer of circular muscles, followed by a layer of longitudinal fibres. The male atrium narrows towards its posterior portion, which receives the opening of the common oviduct (Fig. 9D, E). Then, the male atrium opens into the spacious common atrium that is coated with a thick layer of circular muscles, followed by thick longitudinal muscles in individuals of all the analysed populations (Fig. 9D-F). The gonopore opens ventrally in the common atrium.

The very muscular adenodactyl has a ventral and approximately horizontal position. In individual H0366**,** it consists of a large bulbar part and an elongated papilla (Fig. 9D), similar to specimens H0222 and H0364 from the same population (Majerovo vrilo) (Additional file 12). In the individual H0366, the proximal lumen of the adenodactyl is wide. Interestingly, in specimens H0293, H0294 and H0296 (from the Dretulja River and Dretulja Spring), the papilla of the adenodactyl is shorter. However, the bulb is also very well developed (Fig. 9E). The papilla is rich in longitudinal and circular muscle fibres. The bulb of the adenodactyl consists of intermingled rows of longitudinal and circular muscle that enter the papilla, which is also muscular. A layer of blue circular muscle is visible in the adenodactyl of several specimens (Additional file 12), reminiscent of the “Balkan type of adenodactyl” [107].

### Taxonomic discussion

The species *Dendrocoelum pigmentatum* is distinguished by the presence of dorsal body pigmentation and two eyes. In the genus *Dendrocoelum*, this combination of characters has been reported for *Dendrocoelum superficiale* (Porfirjeva, 1958), the form of *Dendrocoelum lacteum* occurring in the Ohrid Lake area (Kenk, 1978), in *Dendrocoelum lacteum verbanense* (described in [96], and in a set of species endemic to the Ohrid-Prespa Lakes region: *Dendrocoelum albidum* Kenk, 1978; *Dendrocoelum* *cruciferum* (Stanković, 1969); *Dendrocoelum decoratum* Kenk, 1978; *Dendrocoelum dorsivittatum* Kenk, 1978; *Dendrocoelum komareki* (Stanković, 1969); *Dendrocoelum lacustre* (Stanković, 1938); *Dendrocoelum lychnidicum* (Stanković, 1969); *Dendrocoelum maculatum* (Stanković & Komárek, 1927); *Dendrocoelum magnum* (Stanković, 1969); *Dendrocoelum ochridense* (Stanković & Komárek, 1927); *Dendrocoelum presepense* (Stanković, 1969), *Dendrocoelum sanctinaumi* (Stanković & Komárek, 1927) and *Dendrocoelum translucidum* Kenk, 1978.

Regarding the presence of dorsal body pigmentation, it is important to note that *Drendrocoelum* species endemic to the Ohrid–Prespa Lakes share a very similar copulatory apparatus, leading Kenk to use additional traits for species identification -most notably pigmentation. Although variable, he and earlier authors considered pigmentation a recognisable and species-informative character [97, 111–113]. Following this perspective, the use of pigmentation in the diagnosis of other freshwater planarians [114] and recent work on terrestrial planarians [115, 116], we regard adult pigmentation as a useful taxonomic character in this group of species, albeit with appropriate caution. Doing so facilitates comparisons between *D. pigmentatum* and species from the Ohrid–Prespa Lakes and encourages further study of pigmentation in this group of planarians.

Externally, the dorsal pigmentation pattern of *D. pigmentatum* is characterised by a pale band along the midline and a transversal band that forms a pale dorsal cross-like pattern when observed under natural light. This cross-like pattern is absent in *D. superficiale* [117] and in the pigmented forms of *D. lacteum* [96, 97]. Anatomically, a fundamental distinction between *Dendrocoelum superficiale* and *Dendrocoelum pigmentatum* is the absence of a well-developed penis bulb and penis papilla in the former [117]. In the Ohrid form of *D. lacteum*, the penis bulb exhibits an ellipsoidal shape, and the penis presents a true flagellum [97]. In contrast, in *D.* *pigmentatum*, the bulb is not ellipsoidal, and a true flagellum is absent. The shortness of detailed anatomical information for *Dendrocoelum lacteum verbanense* [96] prevents further comparison with *D. pigmetatum.*

Three of the species endemic to the Ohrid-Prespa Lakes region present dorsal pigmentation reminiscent of the cross-like pattern in *D. pigmentatum*: *Dendrocoelum cruciferum*, *Dendrocoelum lacustre* and *Dendrocoelum lychnidicum*. *D. pigmentatum* differs from *Dendrocoelum cruciferum* and *D. lacustre* in that the cross-like bar and the midline are lighter than the rest of the dorsal colour, and a single transverse band forms the cross-like band, while in the former two, it is formed by a pair of rectangular or rounded dark spots or patches [97, 112]. Anatomically, *D. cruciferum* differs from *D. pigmentatum* in presenting a highly developed adhesive organ and a penis flagellum [97, 112]. *D. lacustre* is smaller in size (10 mm in length) than *D. pigmentatum*, and has the eyes in a different position [112]. In contrast to *D. pigmentatum*, in *D. lacustre* the penis papilla is located just anterior to the bulb of the adenodactyl, the bursal canal enlarges at the point of convergence with the common atrium, and the lumen of the adenodactyl is wider [112]. *Dendrocoelum lychnidicum* differs from *D. pigmentatum* in its smaller size (up to 6 mm long) and in having a dorsal colouration of a light chocolate hue that disappears in the head [97, 112]. In contrast to *D. pigmentatum*, *Dendrocoelum lychnidicum* possesses an adenodactyl longer than the pharynx, a penis with weak musculature, a seminal vesicle that is horizontally oriented and receives the opening of the vasa deferentia from the sides, and a very long common oviduct [97, 112].

The remaining *Drendrocoelum* species endemic to the Ohrid-Prespa Lakes region exhibit a pigmentation pattern distinct from that of *D. pigmentatum*. In the case of *D. albidum*, the species differs further from *D. pigmentatum* in its smaller size (9 mm in length) and in possessing a highly developed adhesive organ [97]. In *D. komareki*, the subepidermal musculature is feeble, and the penis papilla is much longer [112] than in *D. pigmentatum*. In *D. prespense*, the penis papilla has a different shape, and the bursal canal enlarges before opening in the atrium (see Fig. 13 in Stanković, 1969) [112]. *Dendrocoelum magnum* is distinguished from *D. pigmentatum* on the basis of its distinctive, bulky body, closer-set eyes, and an almost invariably invaginated penis papilla that is provided with uneven folds [112].

While in *D. pigmentatum* the vasa deferentia open into the lower section of the seminal vesicle, in the following species they open in a different position: *D. dorsivittatum*, *Dendrocoelum maculatum*, *Dendrocoelum ochridense*, *Dendrocoelum sanctinaumi*, *D. decoratum*, *D. translucidum*. Additionally, in the case of *D. dorsivittatum*, the ejaculatory duct is wider than in *D. pigmentatum*, and the common oviduct is characteristically long [97]. In *D. maculatum* and *D. sanctinaumi*, the eye position or head shape differs from that of *D. pigmentatum*, and the penis papilla is longer [45, 46, 97]. In *D. ochridense,* the adenodactyl is longer than the penis [97]. *D. decoratum* is much smaller (8 mm in length) than *D. pigmentatum,* and the penis is longer and with a much wider lumen (Kenk 1978). *D. translucidum* is an almost unpigmented and smaller species (7 mm in length >), with a planariid-like habit and an adenodactyl larger than the penis [97].

Our observations suggest that *D. pigmentatum* bears the Balkan type of adenodactyl. This would be unsurprising given its broad occurrence across *Dendrocoelum*, including Balkan taxa, and would further support the hypothesis that this character is widespread among species from the region [107, 118].

We previously emphasised the limited availability of barcodes for Dendrocoelids (Fig. 1B) and, more generally, molecular data suitable for comparison with *D. pigmentatum*. However, the possible phylogenetic relationship between *D. pigmentatum* and the pigmented *Dendrocoelum* from Ohrid suggested by our analysis (Fig. 4), although limited by the above-mentioned lack of molecular data, underscores the importance of including pigmented dendrocoelids in comparative analyses. In this context, *D. pigmentatum* is at least distinguishable by barcoding from two pigmented Ohrid forms, *Dendrocoelum* cf. *santinaumi* and *Dendrocoelum maculatum* (Figs. 2b, 4). Furthermore, *D. pigmentatum* individuals from the Dretulja River and Spring cluster together in our barcoding analysis, separate from the individual in Majerovo vrilo (Fig. 2b), supporting the existence of interpopulation genetic variability within the species.

Family: Planariidae Stimpson, 1857

Genus: ***Planaria*** Müller, 1776

Species: ***Planaria torva*** (Müller, 1774)

**Material examined**. H0797, Šarena Jezera, Knin, Croatia (44.02686° N, 16.22285° E), 2013, coll. Jochen C. Rink, sagittal sections on 5 slides; H0798, ibid., sagittal sections on 5 slides; H0799, ibid., sagittal sections on 4 slides; H0800, ibid., sagittal sections on 5 slides; H0801, ibid., sagittal sections on 4 slides.

### Taxonomic discussion

**Fig. 10:**
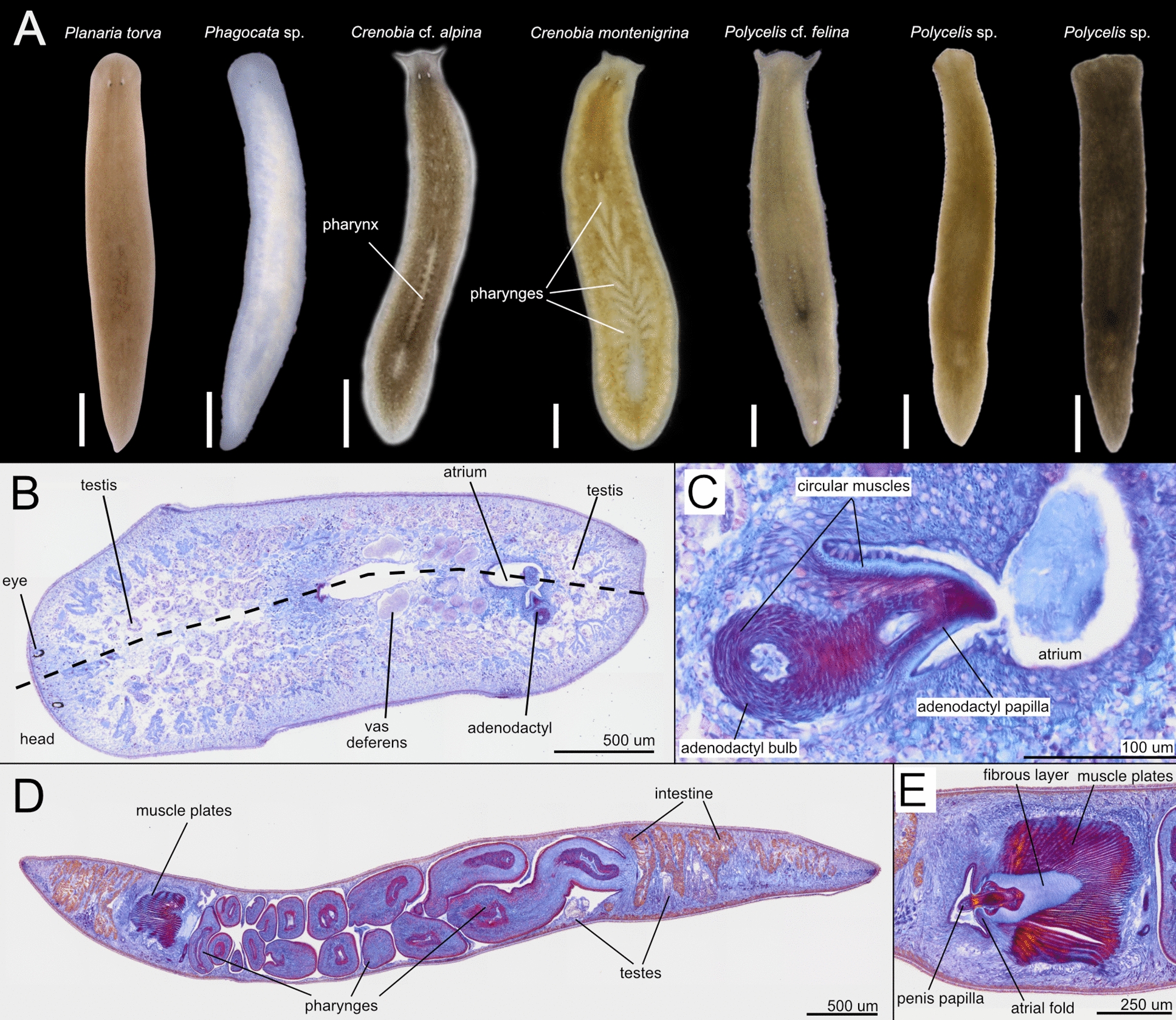
Diversity of planariids. **A.** Living planarian specimens representing the planariids collected during our expeditions. Scale bar, 1 mm. **B-C.** *Planaria torva*. H0798 Brightfield image of horizontal sections. **B.** Testis distribution and adenodactyl position. **C.** Anatomical detail of the adenodactyl. **D-E.** *Crenobia montenigrina*. H0659. Brightfield image of sagittal sections. **D.** Sagittal section showing the presence of multiple pharynxes, the position of the testes, and the muscle plates of the copulatory apparatus. **E.** Brightfield image of the sagittal section of the copulatory apparatus

*Planaria torva* is a widely distributed European species, identified in two Croatian localities based on external morphology and barcoding matches to specimens previously studied and catalogued in our planarian collection. Notably, we found no COI accessions for this species in GenBank, emphasising the need to document its characteristics. The Croatian specimens conform to the species' known external appearance: they measure up to ~ 1.2 cm in length, have a truncated head with two eyes (Fig. 10A), and exhibit a uniform brown dorsal colouration. This description broadly matches descriptions of *P. torva* from the United Kingdom [94] and Herzegovina [95].

Anatomically, *P. torva* is distinguished among European planariids with two eyes by the presence of an adenodactyl, a muscular organ projecting into the atrium. In the Croatian specimens, this organ is visible in histological sections (Fig. 10B-C). This trait, coupled with the barcoding results, confirms the taxonomic designation of these specimens as *Planaria torva*.

Genus: ***Crenobia*** Kenk, 1930

Species: ***Crenobia montenigrina*** (Mrázek, 1904)

**Material examined**. H0659, Vukovicá vrilo, Civljane, Croatia (43.9654° N, 16.4129° E), 22 June 2023, coll. Miquel Vila-Farré, sagittal sections on 10 slides.

### Taxonomic discussion

Sluys recently reevaluated the morphological variability within the genus *Crenobia* [50]. Guided by these findings, we analysed the Croatian *Crenobia montenigrina* specimens to validate their taxonomic assignment. Those specimens exhibit the expected polypharyngeal trait (Fig. 10A, D); the anteriorly shifted mouth opening; large, primarily ventral testes extending posteriorly beyond the root of the first pharynx (Fig. 10D); a pronounced curvature of the sperm ducts; and a thick muscular coat surrounding the atrium (muscle plates) and a fibrous layer characteristic of the genus *Crenobia* (Fig. 10E). In summary, the anatomy of the Croatian specimens is consistent with that of *C. montenigrina * [50].

## General discussion

**Fig. 11:**
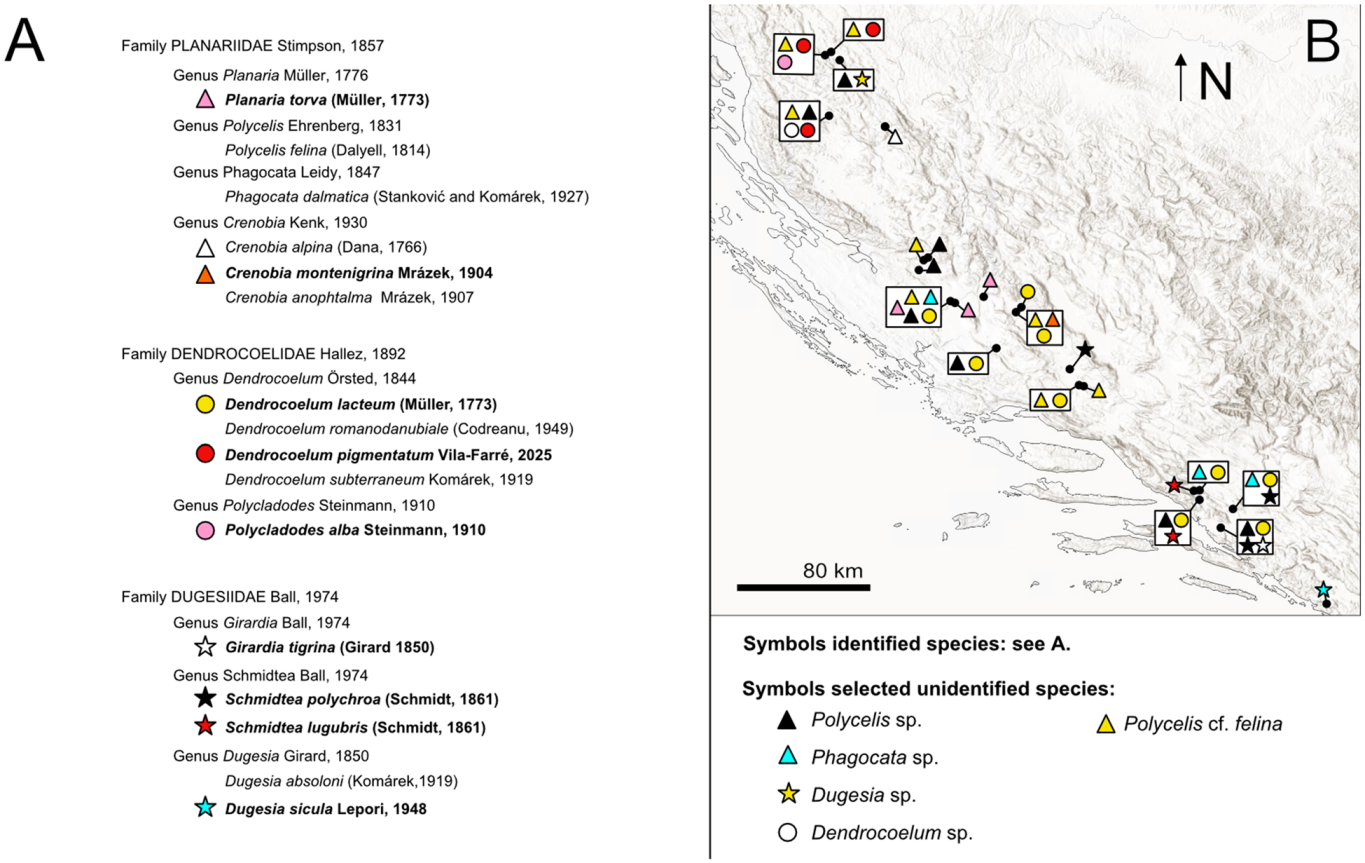
A. Updated list of Croatian Continenticola, excluding terrestrial species. Bold: species studied for this paper. To the left of each species, symbol used in **B** to identify our collection sites for the species. **B.** Distributional records of the species investigated in this study at our collection sites, and of selected samples identified to the genus level

Our sampling in Croatia was limited in scope and restricted to surface waters. Nevertheless, it increased the number of known freshwater planarian species in Croatia from eight to sixteen, with about 35 new planarian records (Fig. 11A, B). While some taxonomically curated records published may have been missed in our bibliographic research, most species cited in the literature are reported from only one or a few localities, making our contribution of multiple sites with planarians more relevant. Given our limited sampling effort, additional sampling in surface waters and caves will likely raise the species diversity in the area.

Our results underscore the importance of integrative approaches in documenting and ultimately preserving biodiversity. The inconclusive barcoding results for specific taxa highlight the limitations of relying solely on COI sequences for taxonomic resolution, particularly when the reference library suffers from taxonomic biases. Notably, COI sequences for common and abundant European freshwater planarians with potentially relevant environmental roles, such as *Dendrocoelum lacteum*, *Planaria torva*, *Polycelis nigra* and *Polycelis tenuis,* are either poorly represented or absent in the current GenBank record. The abundance of representatives from some of these groups in Croatia prompted us to generate new sequences now incorporated into public repositories. These sequences, and additional ones produced in this study from other geographic areas from all over Europe, substantially increase the number of available COI sequences for European planariids and dendrocoelids, contributing to the goal of a taxonomically curated barcoding reference library for planarians.

Our barcoding results were in conflict with the genus assignation of two GenBank barcodes, *Dendrocoelum lacteum* (AF178312) and *Phagocata* (DQ666053) (Fig. 2aC, 2b). In the original publication [119], *Dendrocoelum lacteum* (AF178312) clusters with *Polycelis*, but the COI tree does not include *B. punctacta* or other *Dendrocoelum* species (Fig. 6), a situation later repeated in(99) (see Supplementary data 2D). Interestingly, in the original publication [99], *Phagocata* (DQ666053) is placed next to other planariids in a position coherent with the genus designation (see AF Supplementary data 2D)*.* Given that other *Dendrocoelum* sequences cluster cohesively with different samples of their genus in our analysis, and that other *Phagocata* cluster with *Planaria torva* (another planariid) in a position distant from *Phagocata* (DQ666053) in our Bayesian inference (Fig. 2a, 2b, Additional files 5, 7 A), the taxonomic assignment of DQ666053 and AF178312 in GenBank is very likely erroneous. Further investigation is required, particularly considering the extreme scarcity of *D. lacteum* COI accessions in GenBank.

Our integrative approach further provided new insights into the taxonomy of several taxa. Our investigation of *Schmidtea lugubris* revealed the existence of two genetically distinct lineages, the Central and Adriatic haplotype clades, which were morphologically and karyologically indistinguishable. Even in the case that a more detailed analysis of the two clades should uncover clade-specific differences, our transcriptomic branch-length analysis suggests that the genetic difference between the lineages remains below the level observed between established sister species within *Schmidtea*. This supports the interpretation that the two clades represent intraspecific genetic diversity rather than distinct species.

The geographic distribution of the two major genetic clades in *S. lugubris* is compatible with a hypothetical origin from southern glacial refugia, particularly for the Adriatic clade. Species of Mediterranean origin, isolated in southern European peninsulas during glacial periods, frequently give rise to differentiated genetic lineages. These lineages may remain confined to their respective peninsulas (Balkans, Italy, Iberia) or expand via diverse dispersal routes [120–124]. This pattern is observed in the Balkans across various organisms [124–127], including aquatic species [128–131]. *Schmidtea lugubris* could represent an additional case of a species clade surviving glacial periods in the Balkans, viz., the Adriatic clade. However, due to our limited sampling in much of the Western and Southern Balkans, the precise geographic extent of this Adriatic clade remains uncertain. Further research in the Balkans, Pannonian Basin, and surrounding regions is essential to resolve the species' phylogeography. The presence of highly similar haplotypes in the Central clade across its northern range—from Italy and the United Kingdom to southern Sweden—is compatible with a postglacial expansion from a southern refugium. Additionally, the genetic diversity observed in the eastern part of the Central clade and within the Adriatic clade indicates a complex biogeographic history that warrants further investigation. The biogeography of European planarians inhabiting lowlands (e.g. *S. polychroa*, *D. lacteum*, *P. tenuis* and *S. lugubris*) is barely studied [132], in contrast with cold-adapted species like *Crenobia alpina* [35, 133] or the *Dugesia* in the Mediterranean peninsulas [53, 54, 134]. Our new COI reference sequences and transcriptomes offer an entry point for addressing this gap. Finally, understanding the genetic similarities and differences between the two *S. lugubris* clades deserves further investigation. Modelling the geographic distribution of both clades using species distribution modelling (SDM) software based on occurrence records [134, 135] can potentially identify regions where both clades occur in proximity, which will help clarify the phylogeography and evolutionary history of this recent divergence, e.g. if both clades are in contact or isolated. At the same time, genomic resources for *S. lugubris*(85) and transcriptomes for individuals of both clades can facilitate understanding the genomic differences between the Adriatic and the Central clades (e.g. synteny analysis). Therefore, the complex population history of *S. lugubris* presents an opportunity to explore patterns of planarian genome evolution at the species level.

*Polycladodes alba* is a widespread but rarely studied Centro-European species (see summary in [47]) [136, 137]). Komárek [42] described the cave species *Sorocelopsis decemoculata* Komárek, 1919, based on a single specimen from the cave system Đulin ponor – Medvedica near Ogulin, Croatia. Later, De Beauchamp [46] considered this record an individual of *P. alba.* If *S. decemoculata* is a synonym of *P. alba*, our finding and systematic description of the collected specimens in Croatia mark the species’ reappearance in the country after more than 100 years.

The discovery of a new pigmented *Dendrocoelum* in the Northern Dinaric Karst, far from Lake Ohrid, is striking. Although we cannot rule out human introduction of planarians [138, 139], the presence of genetic differences (COI barcoding tree) and morphological variation (e.g. dorsal pigmentation, anatomy of the adenodactyl, presence of a sphincter) between populations suggests otherwise. The two populations of *D. pigmentatum* in the Dretulja River are separated from those at Majerovo vrilo by a mountain range and belong to two different major drainage systems: the Black Sea and Adriatic basins (see the species description for details).

Most pigmented and a few unpigmented forms of *Dendrocoelum* were formerly assigned to *Neodendrocoelum*, a clade of doubtful taxonomic validity [97, 140]. Interestingly, unpigmented species assigned to this clade at some point have been recorded in localities distant from the Ohrid, e.g. Austria (*Dendrocoelum findeneggi*) [141]*,* Croatia (*Dendrocoelum subterraneum* [111] and, with no taxonomical evidence, *Dendrocoelum plesiophthalmum* [51]), Herzegovina (*Dendrocoelum plesiophthalmum*), or in the Western and Southern Balkans (*Dendrocoelum nausicaae*) [97, 113]. Our phylogenetic analysis, although limited in taxon sampling, is compatible with the existence of a clade of *Dendrocoelum* species widely distributed across the Western Balkans. Hence, our data add an intriguing dimension to the long-debated phylogenetic affinities of these peculiar animals [46, 97, 113, 142], which have proven difficult to resolve by morphological analysis alone [97]. The transcriptomes generated in this study, combined with the description of a new pigmented *Dendrocoelum* from Croatia, provide a valuable entry point for addressing these long-standing questions from a molecular perspective. Additionally, our taxonomic description of the unusually large and charismatic (by planarian standards) *Dendrocoelum pigmentatum* can help in conservation efforts for karstic springs in Croatia.

## Conclusions

The identification of differentiated genetic lineages of *Schmidtea lugubris*, identifying taxonomic ambiguities, and describing new species underscores the need for further research in species-rich areas such as Southeastern Europe. While areas such as the Peloponnesos, southern Ionian islands, the Aegean islands, the Iberian Peninsula and Sardinia have recently been investigated using integrative approaches tools [37, 39, 53], others, such as the southern part of the Dinarides and the Albanids, remain largely unexplored using molecular techniques. Ultimately, our work lays the foundation for a more comprehensive framework for the taxonomy and phylogenetics of European freshwater planarians, particularly in the Western Balkans, and contributes to broader efforts to document and thus conserve aquatic biodiversity. Further efforts are needed to expand molecular and morphological datasets for planarians, particularly for rare and understudied species. Adding multiple planarian COI barcodes from understudied groups represents an entry point for the future generation of a geographically broad and taxonomically inclusive COI barcoding reference library for freshwater planarians.

## Supplementary Information

Additional file 1.

Additional file 2.

Additional file 3.

Additional file 4.

Additional file 5.

Additional file 6.

Additional file 7.

Additional file 8.

Additional file 9.

Additional file 10.

Additional file 11.

Additional file 12.

## Data Availability

All newly reported gene sequences, new raw RNA sequencing, new transcriptome assemblies and histological slides will be submitted to public repositories. Bioproject accession GenBank (RNAseq): PRJNA1289103.
