# Supplementary figures and images for: An integrative taxonomic approach reveals unexplored diversity in Croatian planarians
Source: Front Zool. 2026 Mar 16;23:13. doi: 10.1186/s12983-026-00603-8 (PMC13067409; doi:10.1186/s12983-026-00603-8)

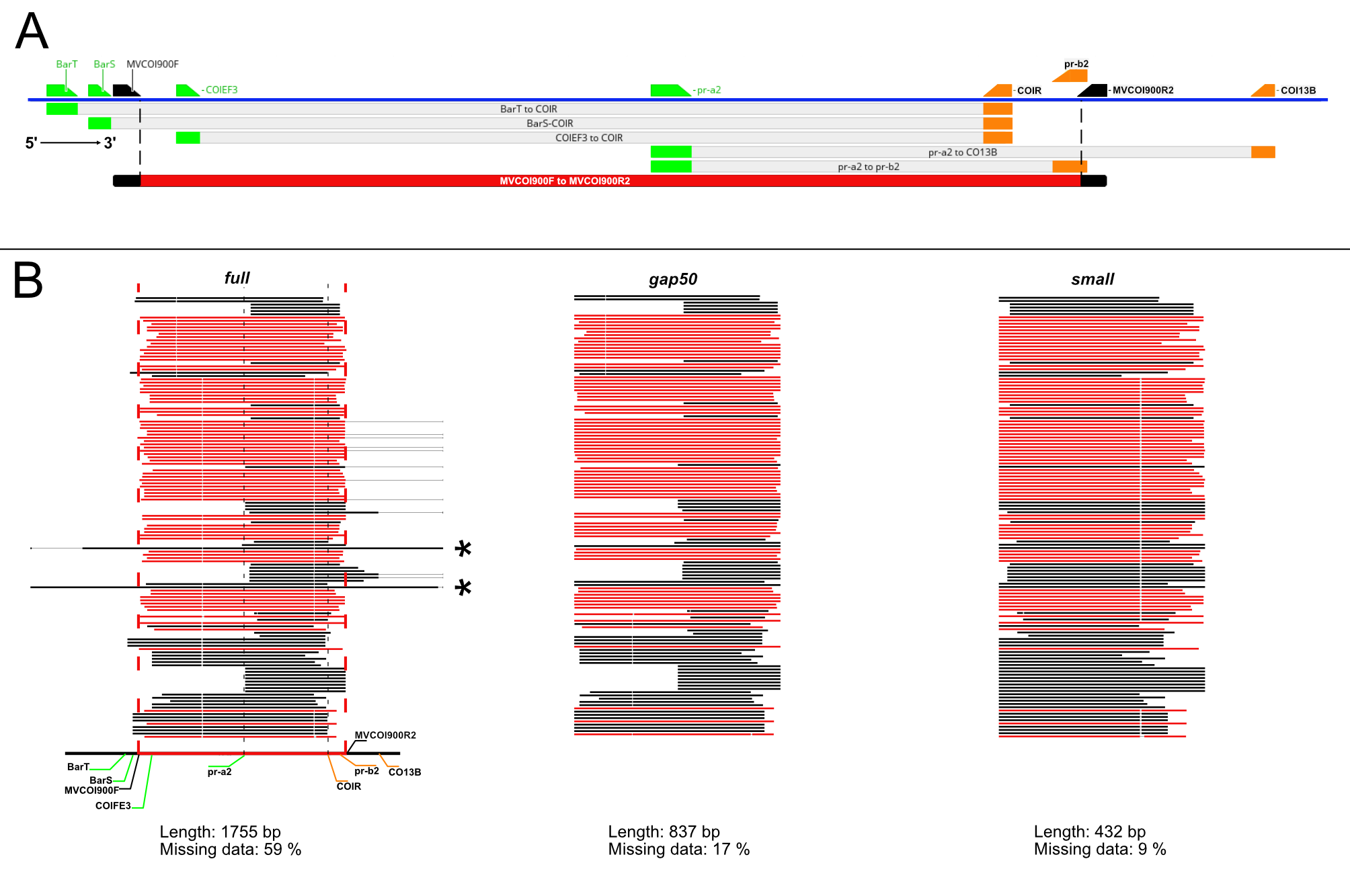

Supplement: Supplementary file 2 — Additional file 2. [file 12983_2026_603_MOESM2_ESM.tiff]

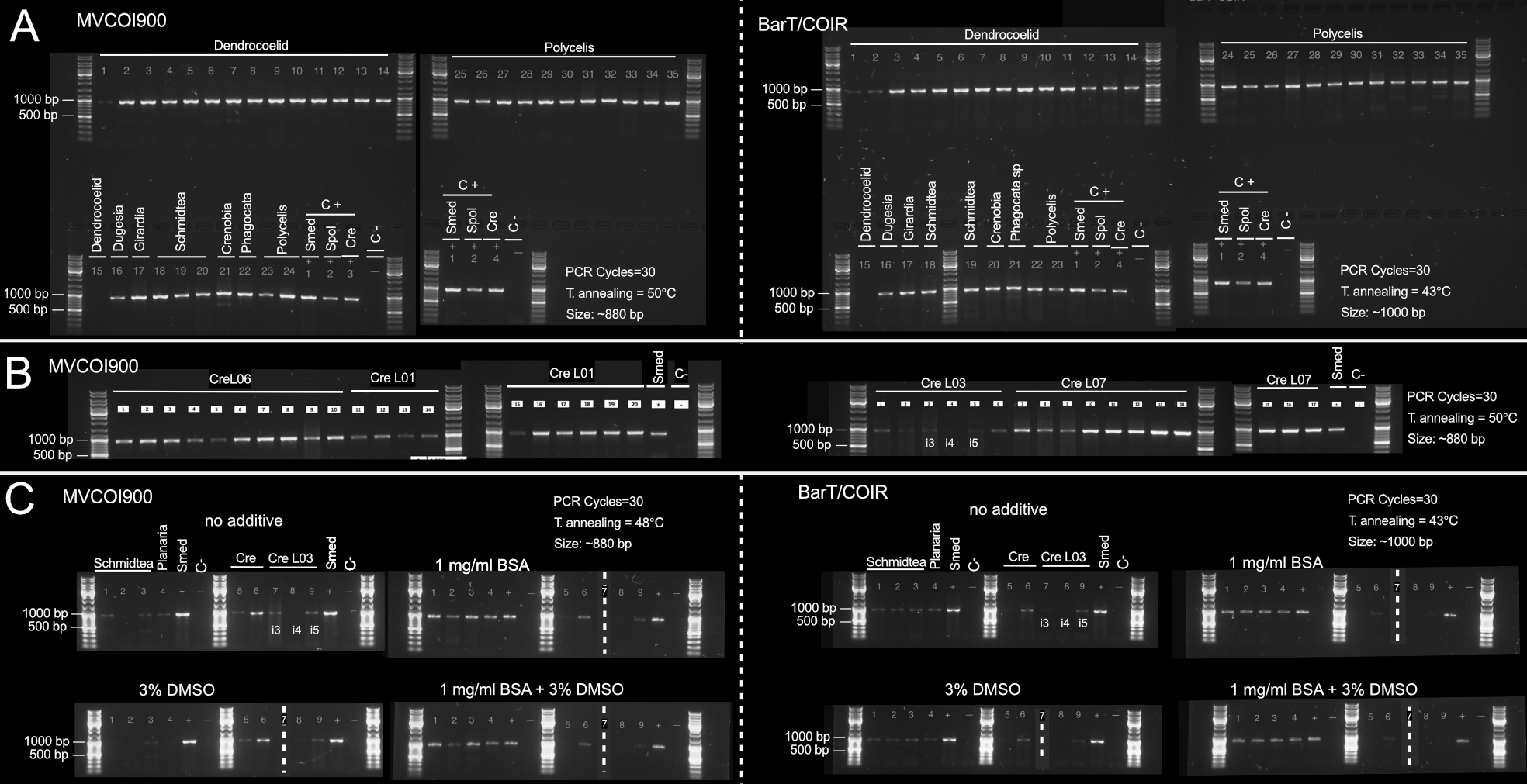

Supplement: Supplementary file 3 — Additional file 3. [file 12983_2026_603_MOESM3_ESM.tiff]

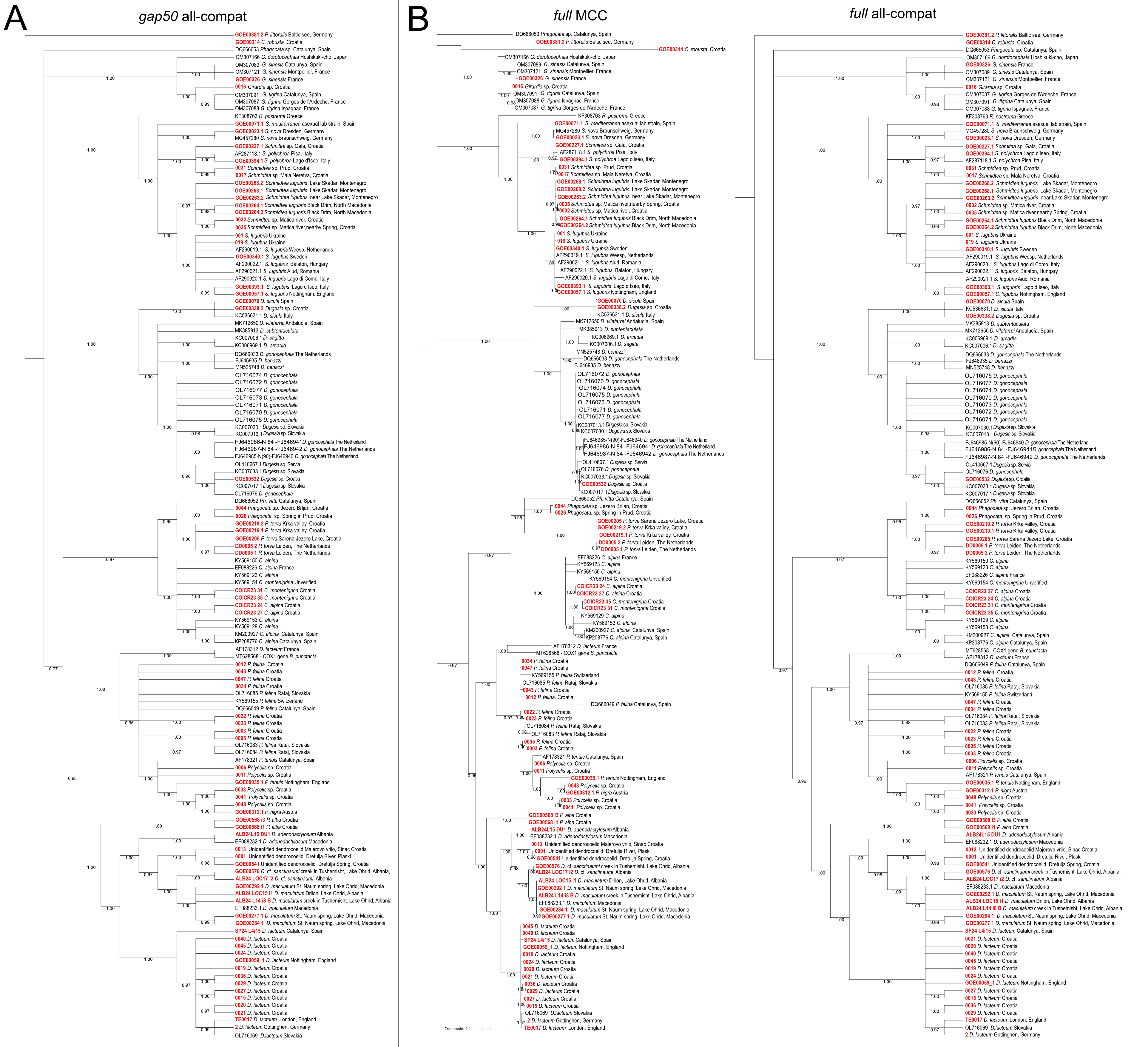

Supplement: Supplementary file 5 — Additional file 5. [file 12983_2026_603_MOESM5_ESM.tiff]

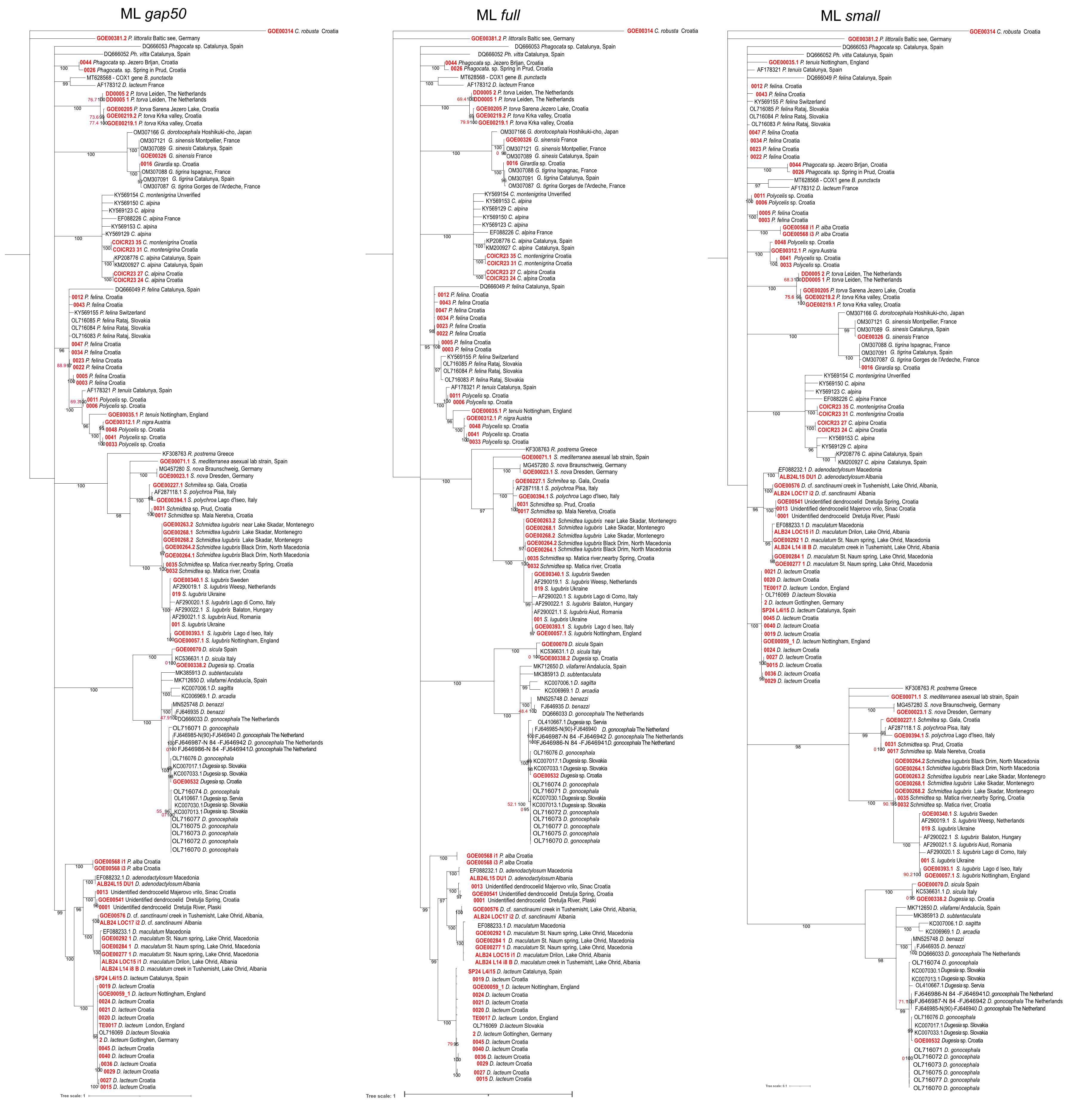

Supplement: Supplementary file 6 — Additional file 6. [file 12983_2026_603_MOESM6_ESM.tiff]

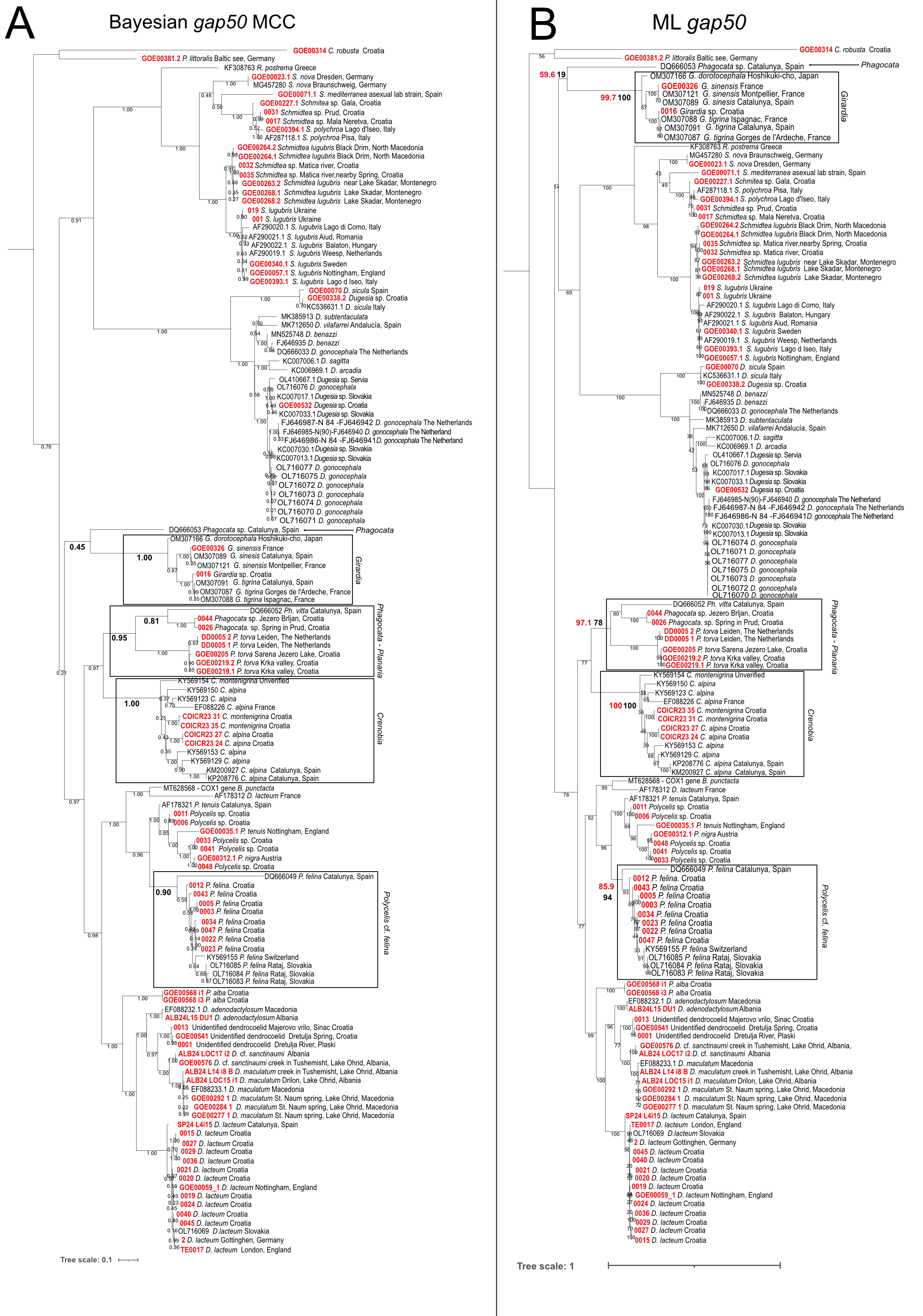

Supplement: Supplementary file 7 — Additional file 7. [file 12983_2026_603_MOESM7_ESM.tiff]

### Bayesian *gap50* versus *full*

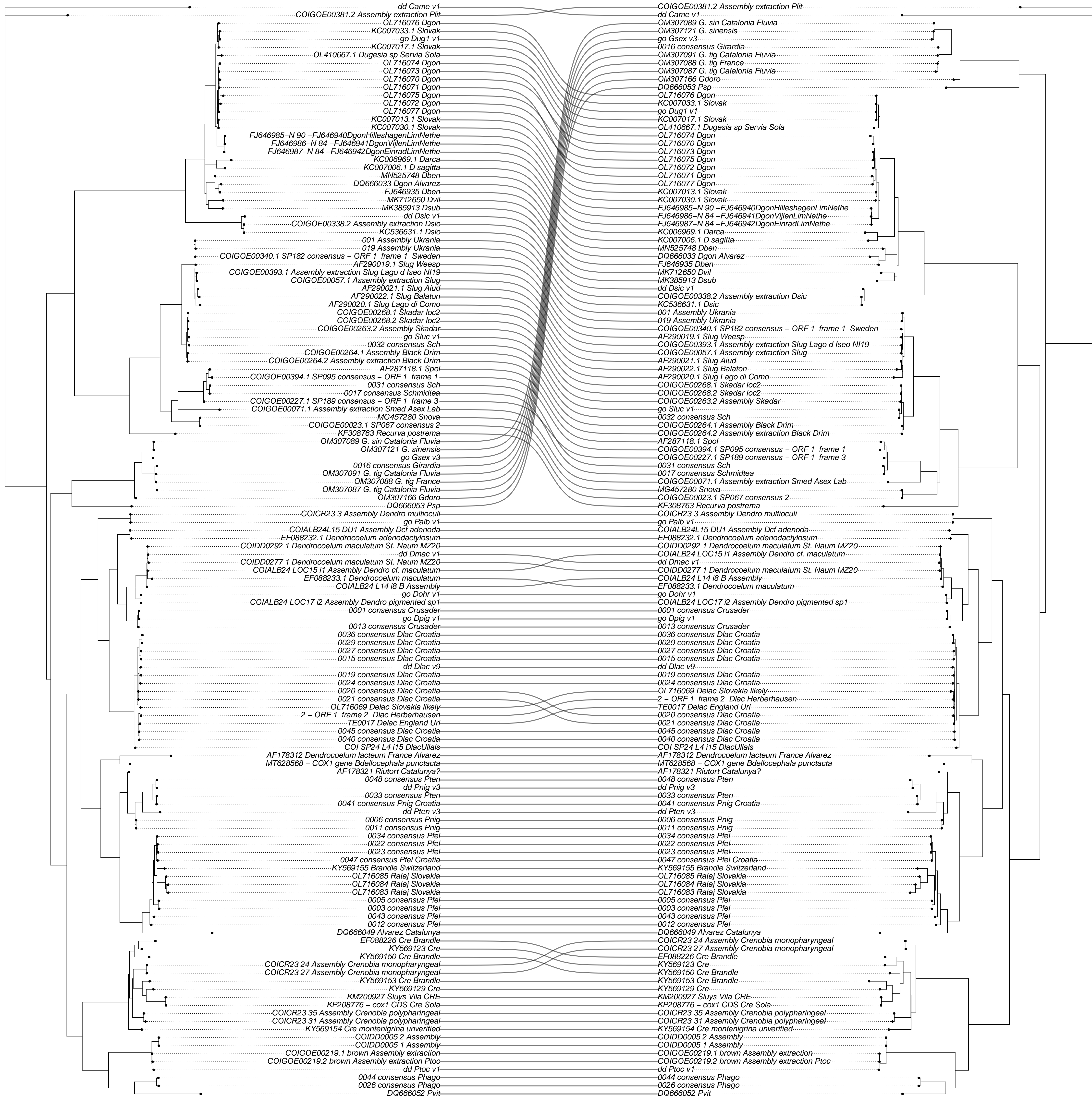

ML full vs gap50

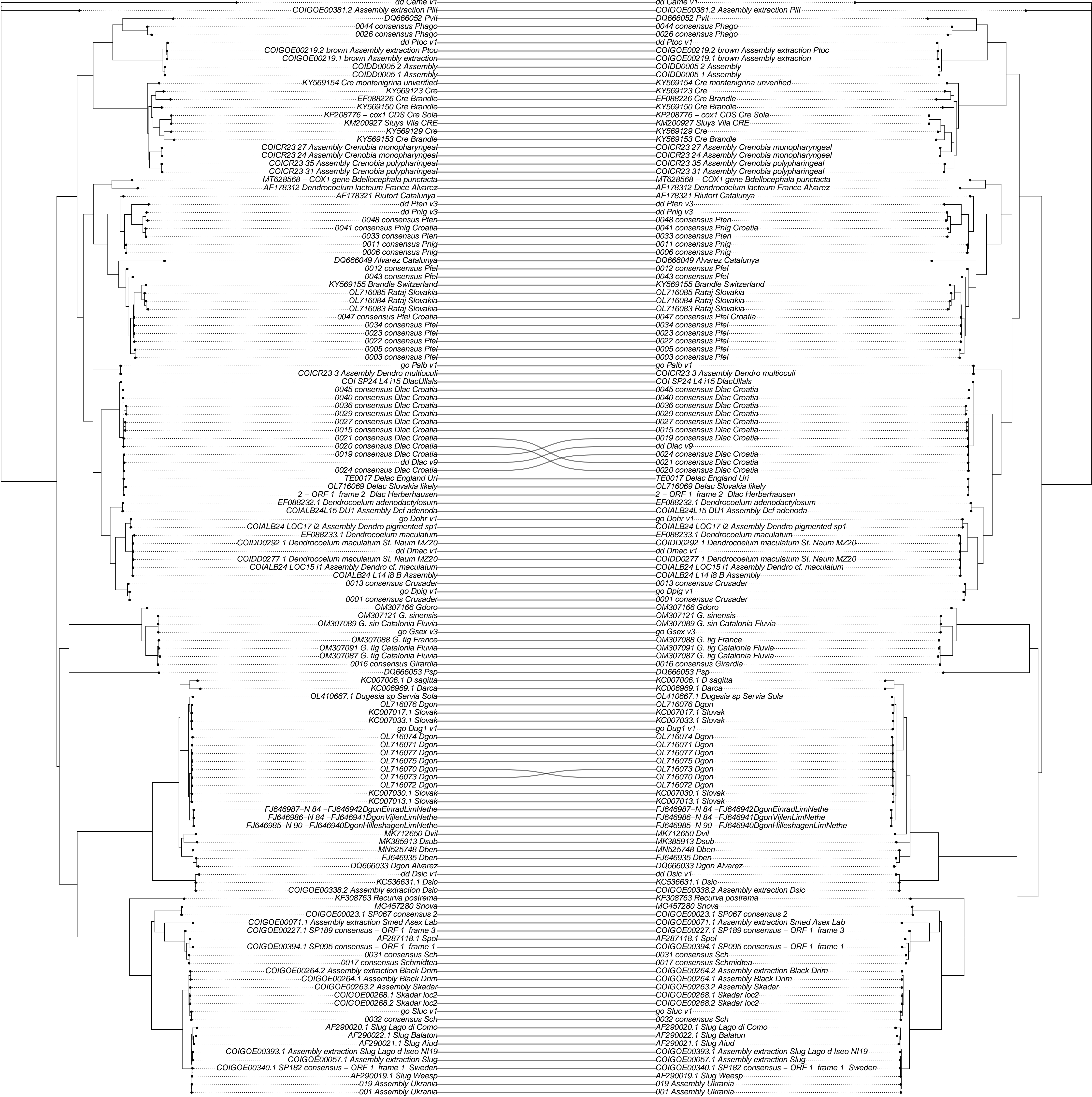

ML versus Bayesian *full*

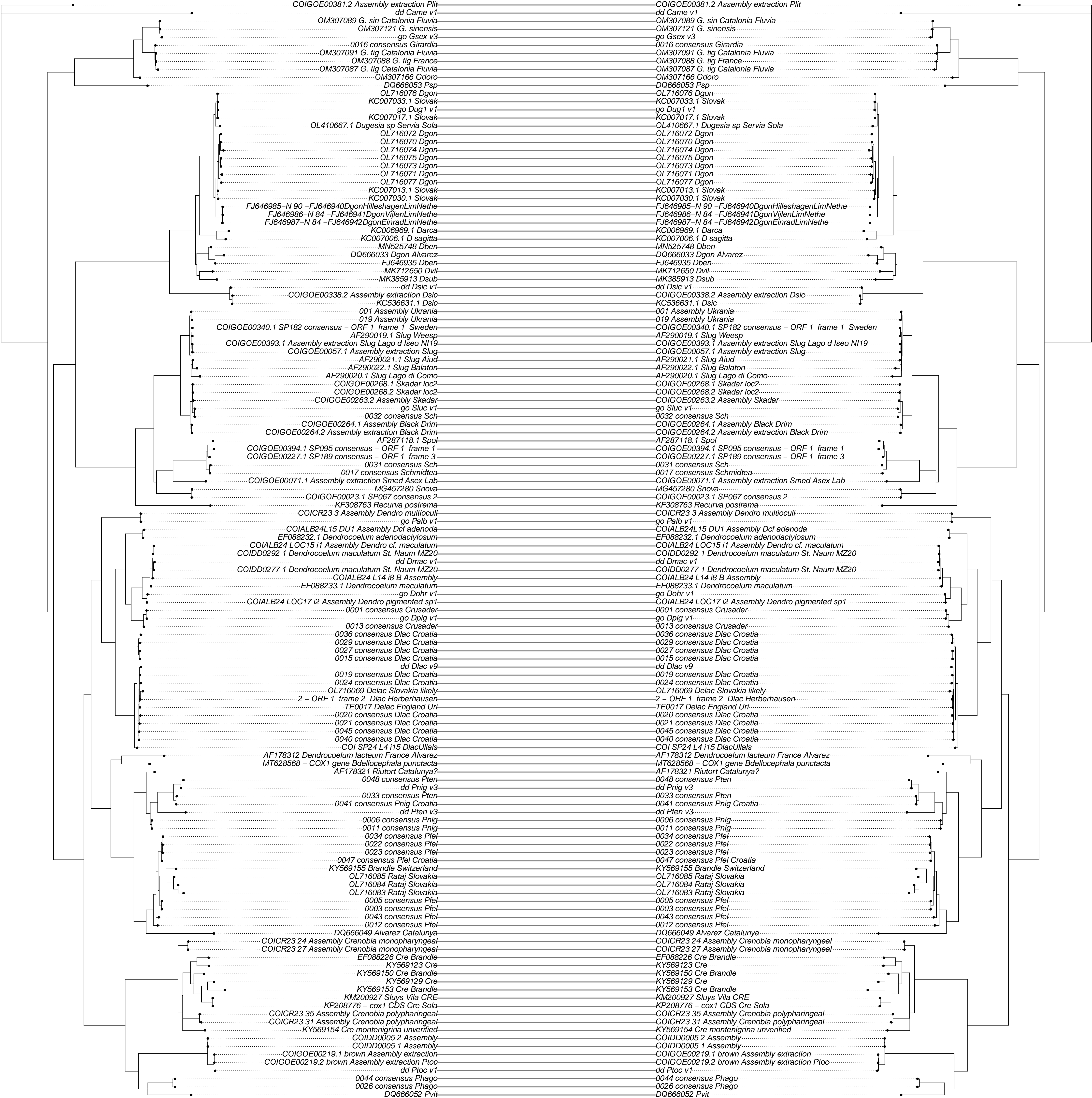

Supplement: Supplementary file 8 — Additional file 8. [file 12983_2026_603_MOESM8_ESM.pdf]

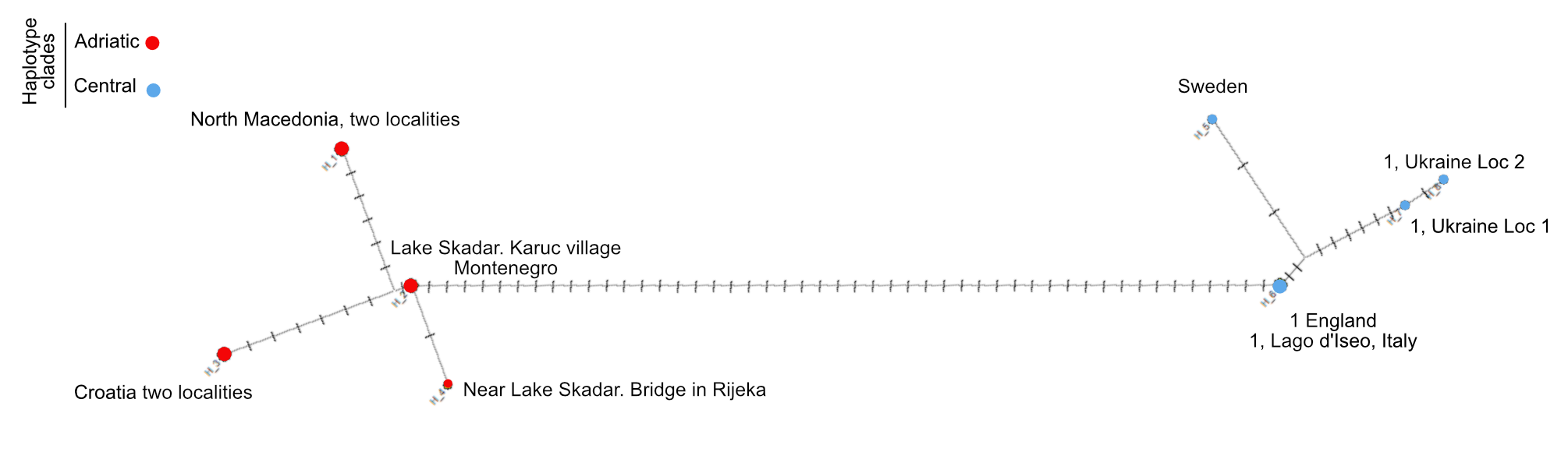

Supplement: Supplementary file 9 — Additional file 9. [file 12983_2026_603_MOESM9_ESM.tiff]

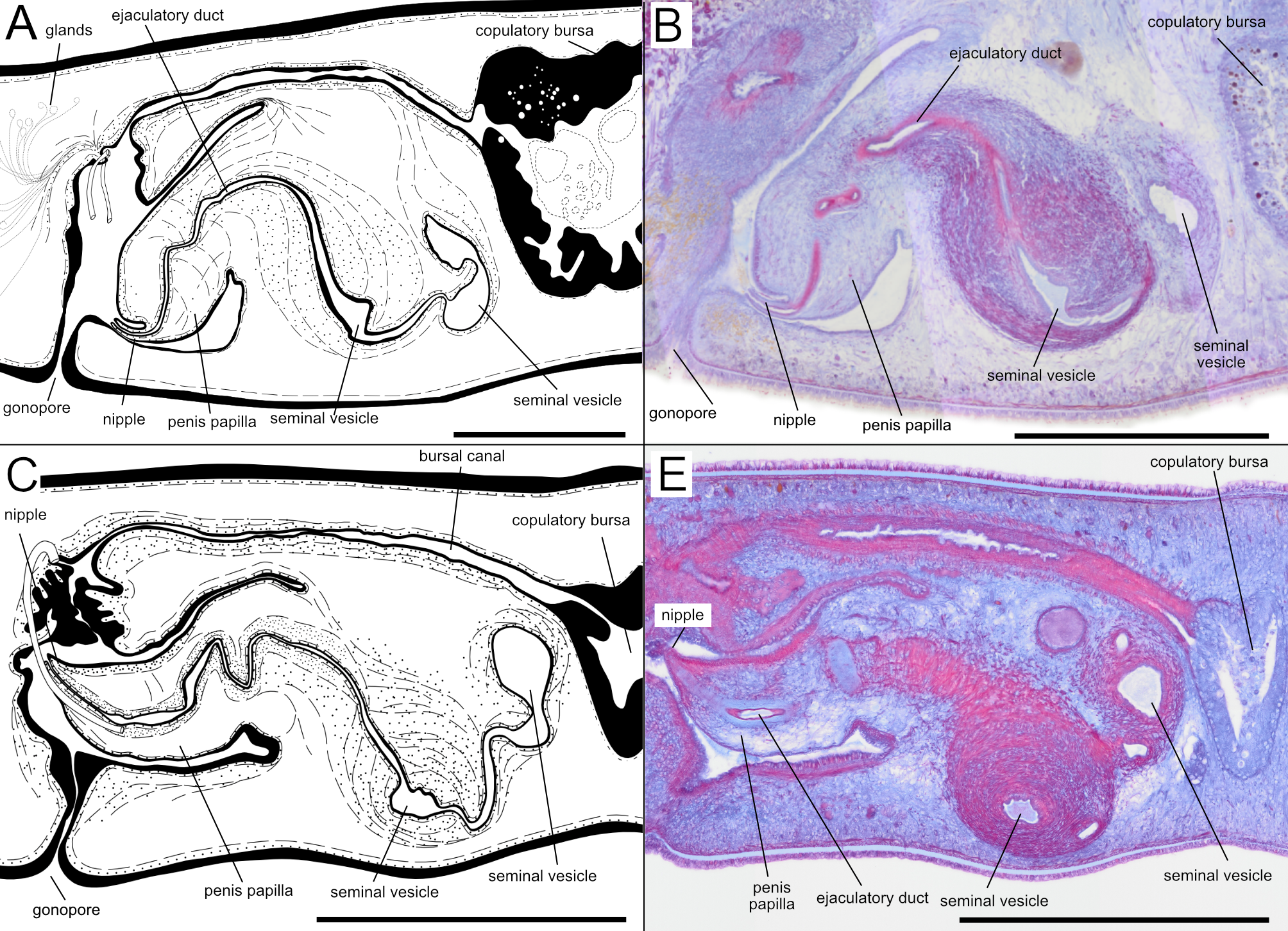

Supplement: Supplementary file 10 — Additional file 10. [file 12983_2026_603_MOESM10_ESM.tiff]

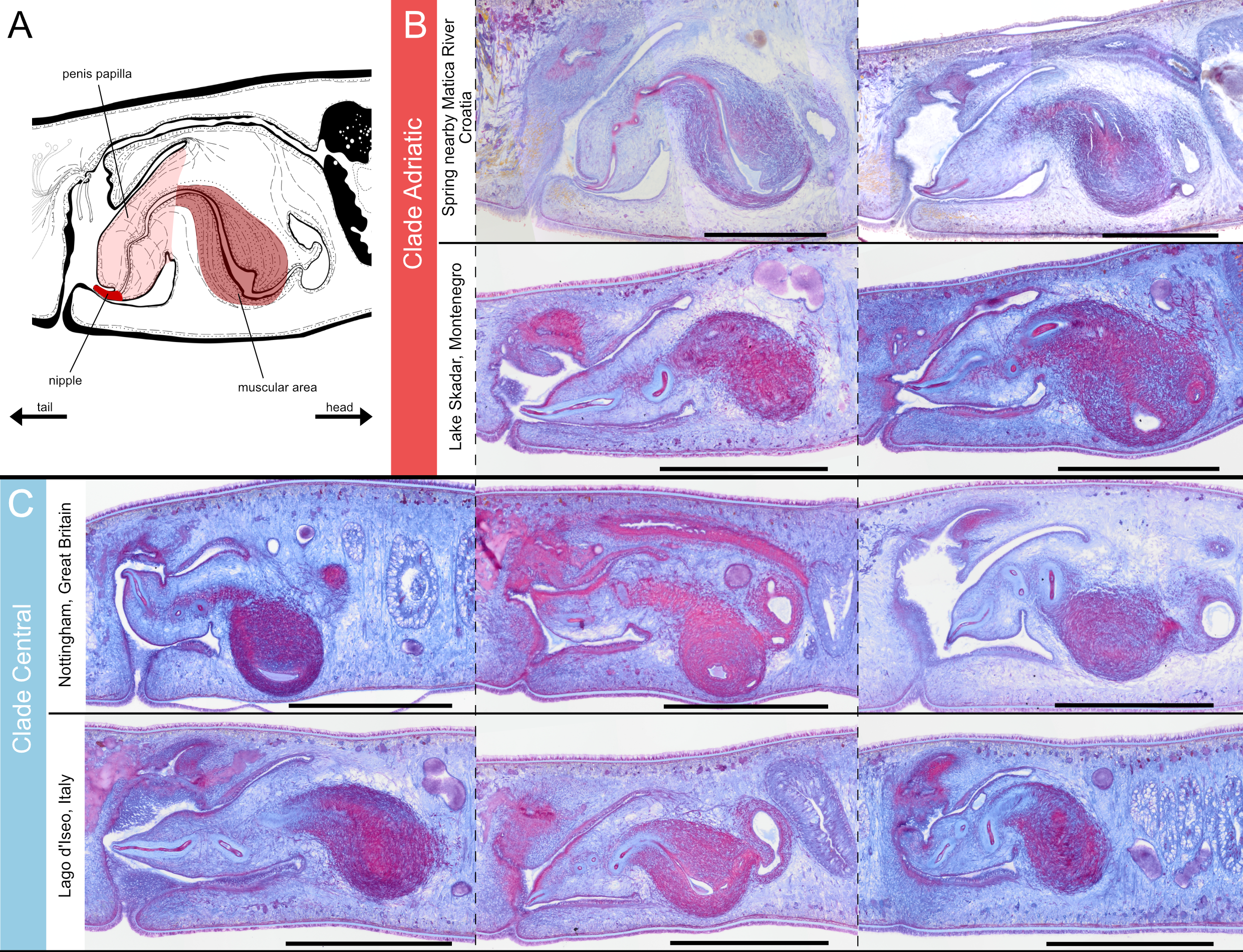

Supplement: Supplementary file 11 — Additional file 11. [file 12983_2026_603_MOESM11_ESM.tiff]

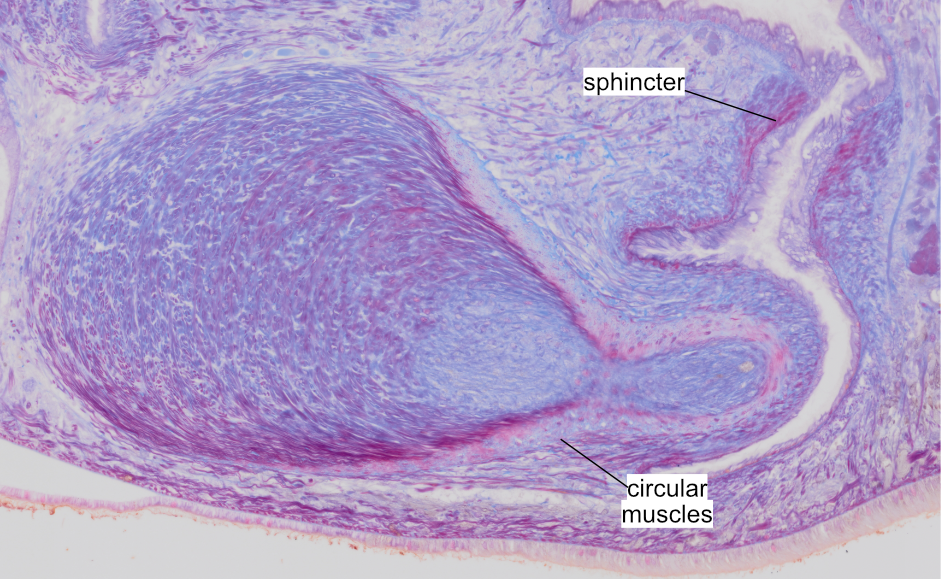

Supplement: Supplementary file 12 — Additional file 12. [file 12983_2026_603_MOESM12_ESM.tiff]
